# The Maya Preclassic to Classic transition observed through faunal trends from Ceibal, Guatemala

**DOI:** 10.1371/journal.pone.0230892

**Published:** 2020-04-07

**Authors:** Ashley E. Sharpe, Takeshi Inomata, Daniela Triadan, Melissa Burham, Jessica MacLellan, Jessica Munson, Flory Pinzón

**Affiliations:** 1 Center for Tropical Paleoecology and Archaeology, Smithsonian Tropical Research Institute, Balboa-Ancón, Republic of Panama; 2 School of Anthropology, University of Arizona, Tucson, Arizona, United States of America; 3 Department of Anthropology, University of Pittsburgh, Pittsburgh, Pennsylvania, United States of America; 4 Department of Sociology-Anthropology, Lycoming College, Williamsport, Pennsylvania, United States of America; 5 Museo Regional de Arqueología la Democracia, Ministerio de Cultura y Deportes de Guatemala, Escuintla, Republic of Guatemala; Universita degli Studi di Milano, ITALY

## Abstract

It is well known that the development of the ancient Maya civilization had significant and long-lasting impacts on the environment. This study assesses a large collection of faunal remains (>35,000 specimens) recovered over a span of several kilometers in and around the archaeological site of Ceibal, Guatemala, in order to determine whether the composition of animal resources was continuous throughout the site’s history between 1000 BC and AD 1200, or whether there were any changes that could be attributed to sociopolitical or environmental causes. Results show a steep uniform decline in the number of freshwater mollusks across the site that occurred during the Preclassic to Classic transition, when large region-wide political changes, including the development of more complex and centralized political organization, took place throughout the Maya region. Evidence of species introductions (e.g., turkeys from central Mexico and possibly the *Dermatemys* river turtle from the Isthmus of Tehuantepec) and variations in resource exchange (e.g. marine shells) over time indicate that Ceibal was one of likely many communities involved in long-distance animal exchange networks. The results of the faunal analysis at Ceibal show how the ancient Maya had a complex and ever-changing relationship with the local wildlife, with outcomes that can still be observed in the environment today.

## Introduction

The application of zooarchaeology, or archaeological faunal analysis, to Mesoamerican research has made great strides in the last few decades as a means of identifying past trends in environmental change, human interactions with the landscape, and sociocultural transitions [1–7]. The variety of animal wildlife in the lowland tropics where the ancient Maya civilization thrived prior to Spanish contact was much greater compared to many other regions of the world that harbored states or comparable centralized polities. Tracking how animal resources were used over time in these communities can reveal how the growth of settlements affected the surrounding wildlife, for the development of large civic centers necessitated new adaptations to the landscape to sustain increasingly large populations [8–11].

**Fig 1:**
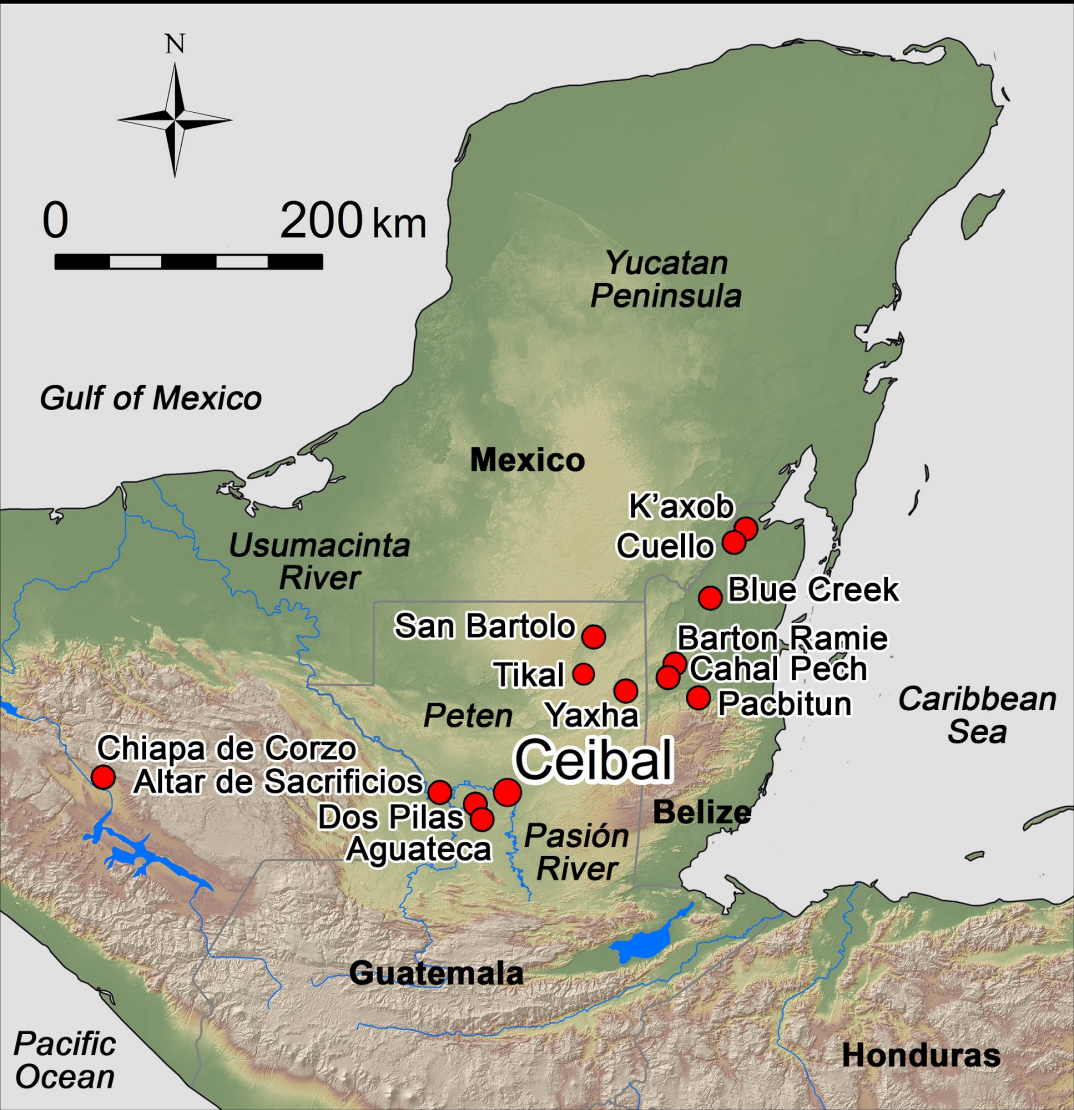
Map of the Maya region, including archaeological sites mentioned in the text. Modified from [16].

This study is the result of a decade of faunal analyses at the Maya community of Ceibal, Guatemala, where intensive excavations have discovered an extraordinarily large quantity of faunal remains. Ceibal was occupied nearly continuously for over two millennia (c. 1000 BC–AD 1200, Table 1), providing a broad temporal range to observe changes in the use of animal resources. While bone preservation is notoriously poor in the humid tropics where Ceibal is located, careful excavation methods that included flotation to recover small faunal fragments, as well as a broad excavation strategy that covered many areas of the site (Fig 1), allowed for the recovery of over 35,000 faunal specimens, most of which were identifiable at least to the taxonomic level of class. Excavations took place across the main central plaza of the ancient urban community, within the monumental central structures and elite residences, and in and around the surrounding residential and minor ceremonial groups, including Caobal, 3 km to the west [12–22]. Such a broad excavation strategy provides information not just regarding how animals were used in different social contexts (for instance, ceremonial activities in the palace court versus dense deposits of trash near an outlying residential group), but also evidence of wide-scale shifts in faunal use that affected the entire region, perhaps even reflecting social and subsistence trends that occurred throughout the greater lowland Maya area.

**Table 1:** The ceramic chronology of Ceibal and the surrounding area.

Period	Ceramic Phase	Years
Early Middle Preclassic	Real-Xe 1	1000–850 BC
	Real-Xe 2	850–775 BC
	Real-Xe 3	775–700 BC
Late Middle Preclassic	Escoba-Mamom 1	700–600 BC
	Escoba-Mamom 2	600–450 BC
	Escoba-Mamom 3	450–350 BC
Late Preclassic	Cantutse-Chicanel 1	350–300 BC
	Cantutse-Chicanel 2	300–150 BC
	Cantutse-Chicanel 3	150–75 BC
Terminal Preclassic	Xate 1	75 BC–AD 50
	Xate 2	AD 50–125
	Xate 3	AD 125–175
Early Classic	Junco-Tzakol 1	AD 175–300
	Junco-Tzakol 2	AD 300–400
	Junco-Tzakol 3	AD 400–500
	Junco-Tzakol 4	AD 500–600
Late Classic	Tepejilote-Tepeu 1	AD 600–700
	Tepejilote-Tepeu 2	AD 700–750
	Tepejilote-Tepeu 3	AD 750–810
Terminal Classic	Bayal	AD 810–950
Postclassic	Samat	AD 1000–1200

This large dataset, spanning two millennia, allows us to address the following questions: Do we see any major changes in the use of certain animals over time? Do specific categories of taxa appear or disappear over time, perhaps signifying the introduction or extirpation of certain species? Do changes reflect known political and economic shifts in the region, indicating that at least some taxa were affected by human social trends? Do we see shifts that affect the entire site as a whole, or only certain areas of the site (i.e., ceremonial core vs. outlying residential groups), the latter indicating that fauna acquisition and use may have been restricted in some areas and not in others? Or is there no significant change at all over time?

To answer these questions, this study compares large-scale trends in the use of fauna across all excavated contexts at Ceibal’s ceremonial core, its outlying residential and minor ceremonial groups within a 2 km^2^ range, and the minor ceremonial center of Caobal about 3 km to the west of Ceibal’s core. The abundance and diversity of taxa are compared among different areas over time, in order to assess whether or not there is a significant change in animal resource use, either at the site as a whole or within specific areas. Trends in the data are compared with the growing number of zooarchaeological reports from other sites in the Maya region (Fig 1), to determine if trends are unique to Ceibal, or are signs of larger region-wide shifts in fauna use over time, possibly due to far-reaching political, economic, or even environmental changes.

### Background to the study site

**Fig 2:**
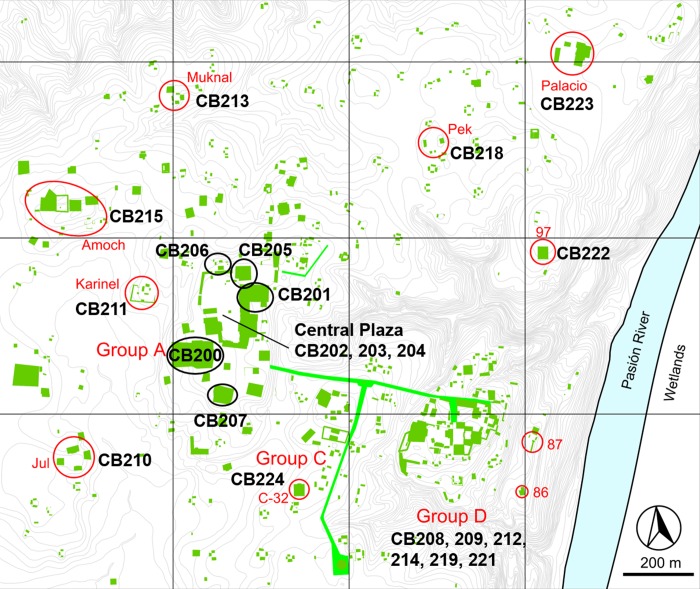
Map of Ceibal, including excavation locations and operation numbers. CB216, CB217, CB220, and CB225-228 are not shown. Map modified from [16].

Ceibal, also known as Seibal, was a large Maya community located on the banks of the Pasión River, a tributary of the larger Usumacinta River that flows north to the Gulf of Mexico. Its many well-preserved stone stelae and other monuments attracted art historians and archaeologists in the late nineteenth century, and eventually Harvard University began an extensive survey and excavation project at the site in the 1960s, directed by Gordon Willey [23–25]. Harvard archaeologists investigated various parts of Ceibal, including Groups A, C, and D (Fig 2), and surrounding residential areas, but their excavations focused on Late and Terminal Classic period (c. AD 600–950) components. Substantial Preclassic layers buried under Classic period structures were not fully explored. A second series of excavations was conducted by the Ceibal-Petexbatun Archaeological Project (CPAP) from 2005–2017, made up of an international collaboration of researchers that included Takeshi Inomata and Daniela Triadan (University of Arizona), Kazuo Aoyama (University of Ibaraki), and Flory María Pinzón, Eric Ponciano, Victor Castillo Aguilar, and Juan Manuel Palomo (Institute of Anthropology and History of Guatemala and Universidad de San Carlos, Guatemala). Jessica Munson directed excavations at Caobal, a minor ceremonial center west of Ceibal’s ceremonial core. Melissa Burham directed excavations at the Amoch, Jul, Muknal, Palacio, and Pek Groups, a mix of elite residential and minor ceremonial groups surrounding the Ceibal monumental core. Jessica MacLellan directed excavations at the residential Karinel Group, west of Group A. The CPAP excavations covered a broad region of the site and its outlying communities, and also focused on Ceibal’s early history, in a time when Ceibal was one of the few early monumental centers in the Maya area [14, 15, 26].

Extensive excavations across Ceibal have corroborated that people began living relatively permanently at the site during the early part of the Middle Preclassic period (c. 1000 BC), when ceramic technology began to become increasingly widespread throughout the lowland Maya region [14, 15, 27]. Ceibal was one of the first lowland Maya communities to build monumental structures with both earth and stone, massive enterprises which would have required the collaboration and direction of many people. Many of these early projects, which were later built over by subsequent generations, are located in Ceibal’s Group A, a large artificial plateau with structures rimming a Central Plaza that was used for millennia [26]. Ceibal’s population likely numbered in the hundreds or thousands by the end of the Middle Preclassic period (c. 400 BC). It experienced a decline in population, and likely political instability as well, in the period known as the Early Classic (c. AD 250–600). A substantial part of the center was abandoned between AD 500 and 600.

Around AD 600, the population of Ceibal began to recover. Groups A and D served as primary foci of elite activities [12]. After a defeat by Dos Pilas in AD 735, Ceibal became a vassal community for a time until Dos Pilas suffered a military defeat. A possibly illegitimate ruler took control after AD 770. By the start of the Terminal Classic period around AD 810, Ceibal had fallen into a period of decline and social upheaval. The arrival of a foreign dynasty enabled a resurgence to power around AD 829 when Ceibal finally became a capital center, but the site was completely abandoned around AD 900 or 950, following similar abandonments throughout the lowland region. A small population returned to the area between AD 1000 and 1200 [16], and the site was permanently abandoned thereafter.

Ceibal’s long history, including two distinct rises to social prominence, provides an excellent opportunity to track the use of animal resources over time. A recent study [28] on the marine mollusk taxa recovered from Ceibal’s 2005–2016 excavations revealed that there were distinct trends in which marine taxa were traded and used for ornamentation at specific times, suggesting that region-wide socioeconomic circumstances strongly influenced what fauna were or were not imported to the site. Faunal analyses of the Harvard excavation animal bones, conducted by Mary Pohl [29–31], found that Ceibal’s Classic period faunal assemblage resembled patterns found at other lowland Maya urban centers, including a focus on white-tailed deer (*Odocoileus virginianus*) among the Maya elite. Whether these status-based distinctions were present prior to the Classic period, when Ceibal was an early monumental center during the initial formation of a Maya state in the Preclassic, is one of the foci of the present study. This study also examines the potential environmental impact the Maya may have had on the local faunal community, which may be observable over a period of two millennia.

## Methods

### Recovery in the field

All faunal specimens reported in this study were recovered from the CPAP excavations conducted during the 2005–2017 seasons. Excavations were carried out in a number of operations across the Ceibal ceremonial core and its peripheries, as well as the nearby smaller ceremonial center of Caobal. Excavations were conducted in the same manner by all archaeologists across the site with frequent supervision by project directors, using primarily culturally-determined stratigraphic sequences (i.e., levels and lots were separated depending on changes in the sequence of construction, such as plaster floors within a structure or plaza, as well as special features like burials or cache deposits). Sample recovery strategies were therefore largely consistent across the site. Archaeologists at Ceibal were careful to recover all faunal specimens when encountered, even potentially intrusive taxa such as terrestrial snails. Soil from every excavation was sieved with a ¼-inch screen mesh. Project archaeobotanist Hiroo Nasu (Graduate University for Advanced Studies, Japan) performed soil flotation with water and 2 and 4-mm sieves with ~1-liter samples collected from many deposits across the site; the heavy fractions were separated by Sharpe. This process resulted in the collection of many tiny specimens that would otherwise be lost using a traditional ¼-inch screen.

### Faunal analysis

Two export permits were granted from the Instituto de Antropología e Historia de Guatemala (Guatemalan Institute of Anthropology and History) in order to identify archaeological faunal specimens in laboratories in the USA and Panama: DAJ-DGPCYN/196/2013 (in 2013 for the USA) and DAJ-299-2017 (in 2017 for Panama). Sharpe identified all faunal specimens from the site between the years 2010–2019. Due to the large quantity of faunal specimens and exportation limitations for archaeological specimens from Guatemala (one box of specimens per exporter at a time), many specimens were identified in the CPAP laboratory in Guatemala City, Guatemala. These were the specimens most easy to identify and heavy to transport, particularly invertebrates like the freshwater mussels. CPAP human osteologist Juan Manuel Palomo (University of Arizona) assisted in the separation of mixed human and animal fragments. Two large sets of specimens were exported for analysis with relevant comparative collections at the Florida Museum of Natural History (Gainesville, FL, USA) and the Smithsonian Tropical Research Institute (Panama City, Panama).

Taxonomic identifications are currently in flux due to rapid developments in molecular biology [32]. The vast majority of specimens were identified to the level of class, and whenever possible, attempts were made to identify each specimen to the most specific rank possible. Taxonomic terminology was checked with the Darwin Core glossary of standards [33] to ascertain that all terms were currently accurate, with assistance from the Integrated Taxonomic Information System [34] and the World Register of Marine Species [35]. Some taxonomic terms are still unresolved (for example, Staurotypidae [36]), which are indicated in our species list.

Due to the wide variety of taxa in the area around Ceibal near the Pasión River, the taxonomy of certain species remains ambiguous. This can be explained by two factors: first, the phylogenetic relationship and taxonomic name of some species are not yet known, and second, no comparative skeletal or shell collection exists that has an example of every species from the Pasión region. The first factor mainly affects invertebrates, particularly freshwater mollusks and terrestrial gastropods [37–39], as well as some of the fish species (especially cichlids [40, 41]). While studies are underway to clarify the taxonomic designations for some of these (e.g., [42] and [43], a phylogenetic assessment of the modern freshwater mussels, Unionidae, around Ceibal), the present study uses conservative nomenclature and identifies such specimens to the level of least ambiguity (e.g., freshwater mussels are all considered Unionidae). The second factor, regarding the lack of an extensive faunal collection to make identifications of all Pasión-specific fauna, results in incomplete identifications for species-rich classes, such as fish and birds. Flotation methods recovered many small fish bones, most of which were identified to the level of ray-finned fish (Actinopterygii) for this study. Many of these fish have identifiable elements, and identification is ongoing. Almost every bird bone was exported for comparison with a skeletal collection. However, although identifications could be made in many instances to the level of family or even genus, no comparative collection has every bird species in the tropics, so many taxa were tentatively identified to the closest match in a collection (if they could not otherwise be identified with consultation from an expert). Such specimens are preceded by the designation “cf.” (Latin *confer*, or most comparable with).

Specimen quantification strategies have been hotly debated over the last several decades in zooarchaeology [44–54]. In short, there is no single quantification method that works well at every site, nor is every technique equally applicable to solving every question. The bones and shells of different taxa break or erode in different ways over time in the ground, and different preservation conditions either improve or worsen the effects of such breakage. Furthermore, in the case of ancient Maya society, secondary midden material was often used to construct structures or patios and was spread out; this leads to a single broken bone being separated across a wide distance. For example, several bones and shells that could be refit were recovered a few meters apart in the Group A Central Plaza as well as the Karinel Group. There are also areas of Ceibal that exhibited a considerable amount of mixing between levels, especially between Late and Terminal Classic phases near the surface. The chronological periods were assigned based on ceramic phases determined by the project archaeologists, as well as direct radiocarbon dates on animal bones and other organic material in the excavation lots. Thus, while it is probable that some taxa in this study have been associated with the wrong period due to this mixing problem, every effort has been taken to determine the most likely date. Faunal specimens that could not be associated with any period, usually because of severe mixing between levels, were not included in this study, although these specimens are reported in the Supporting Information section (S1 Table).

While most individual fragments (NISP, or number of individual specimens) were counted in this study, if bones could be refit into an individual element or otherwise displayed characteristics of belonging to the same element (e.g., five fragments of a deer humerus that were clearly the same bone), these are reported as a single specimen in the results. A record of split fragments counted as single specimens, as well as evidence of recent breaks, can be found in the Supporting Information (S1 Table under the column labeled “Comments”). Comprehensive counts of all fragments overquantify specimens that break easily, such as armadillo shells or large mammal bones. Comprehensive NISP counts are used in this study to quantify unidentifiable fragments that do not refit (e.g., unidentifiable mammal long bone shaft fragments), but are not used to quantify identifiable elements (e.g., a fragmented deer humerus). Thus, the NISP reported for all animal classes in this study is a conservative count of refitted remains. Armadillos and turtles have two NISP counts in the result tables, the first being the total number of fragments and the second in brackets designating the lowest number of shell elements in the excavation lot (e.g., 50 turtle non-overlapping carapace fragments found in one lot is considered one carapace, or 50 (1)). Invertebrate shells frequently shattered and were never quantified by individual fragment; rather, bivalves were quantified to least number of individual valve per lot (e.g., three left-sided Unionidae valves), and gastropods were quantified to the least number of individuals, usually based on interior whorls (for instance, large concentrations of apple snails (*Pomacea flagellata*) were always quantified by the number of shells exhibiting interior whorls, which were the most diagnostic and preservable part). Shell ornaments were counted as individual specimens (NISP = 1).

The minimum number of individuals (MNI) within each taxon was also calculated. MNI is useful for determining a more realistic proportion of different taxa at a site. Yet MNI is a complicated number to determine, particularly in the Maya area where it is possible that one skeleton has been scattered as secondary midden material within construction fill across a wide area. Toward this end, the site was subdivided into regions where it was most likely that the same animals would be found (S3 Table). For example, excavations in the Karinel Group were fairly close to one another [17, 18], and so the entire residential group was considered a potential area for finding the same individual skeleton. In addition to spatial and temporal differences, MNI was calculated using the greatest number of repeating same-sided elements in an individual (for example, five right-sided deer humeri would be an MNI of five deer). Age differences among animals, such as degree of bone epiphyseal fusion, were also considered.

Taxonomic diversity, or the relative importance of species at a site, was compared using the Shannon–Weaver formula as described in by Reitz and Wing [55]:

$$H'=-\sum \left(p_{i}\right)\left(log_{e}p_{i}\right)$$ (1)

Diversity (H’) is governed by the relative importance of each taxon in an assemblage, or richness, in addition to how evenly individuals within taxa are distributed in an assemblage, or equitability. H' is considered the Shannon-Weaver diversity index value, and p_i_ is the relative abundance (MNI) of individuals for each taxon in the assemblage. Higher H' values are more diverse than lower values. The most highly resolved taxonomic levels were used for this analysis to avoid overlapping categories; thus, general categories like “unidentified mammal” were excluded.

Equitability was measured using the formula:

$$V'=H'/log_{e}S$$ (2)

Here, V' is the equitability value, H' is the Shannon-Weaver diversity index, calculated for the sample in question, and S is the number of taxonomic categories for which the MNI values were derived. Equitability values range from 0–1.0, with 1.0 representing an even distribution of taxa, whereas 0 is the most unevenly distributed, meaning all specimens belong to a single taxon.

Since this study is focused primarily on assessing the overall distribution of fauna at Ceibal and how and why it changed over time from an ecological and social perspective, it does not address more detailed topics such as human-modified artifacts like tools and ornaments, nor does it assess activity areas, ritual deposits, and other location-specific issues. These are important topics within zooarchaeology, and will be the focus of future works in development. A detailed account of the marine invertebrates has been reported previously [28], and so marine trade will only be briefly discussed in this study as it pertains to the overall trends.

## Results

### Overall results

Faunal remains came from nearly every excavation unit at the site (Fig 2; see S1 Table for specific lot information). Although most units had both Preclassic and Classic phases represented, fauna recovered at Group D dated almost exclusively to the Late and Terminal Classic periods. The animal remains from most operations were recovered from construction fill deposits, perhaps as secondary or tertiary midden material, with the exception of a dense deposit of Early-Late Classic material on top of Str. A-2 and Late and Terminal Classic primary middens in the Karinel residential group and Group D.

**Fig 3:**
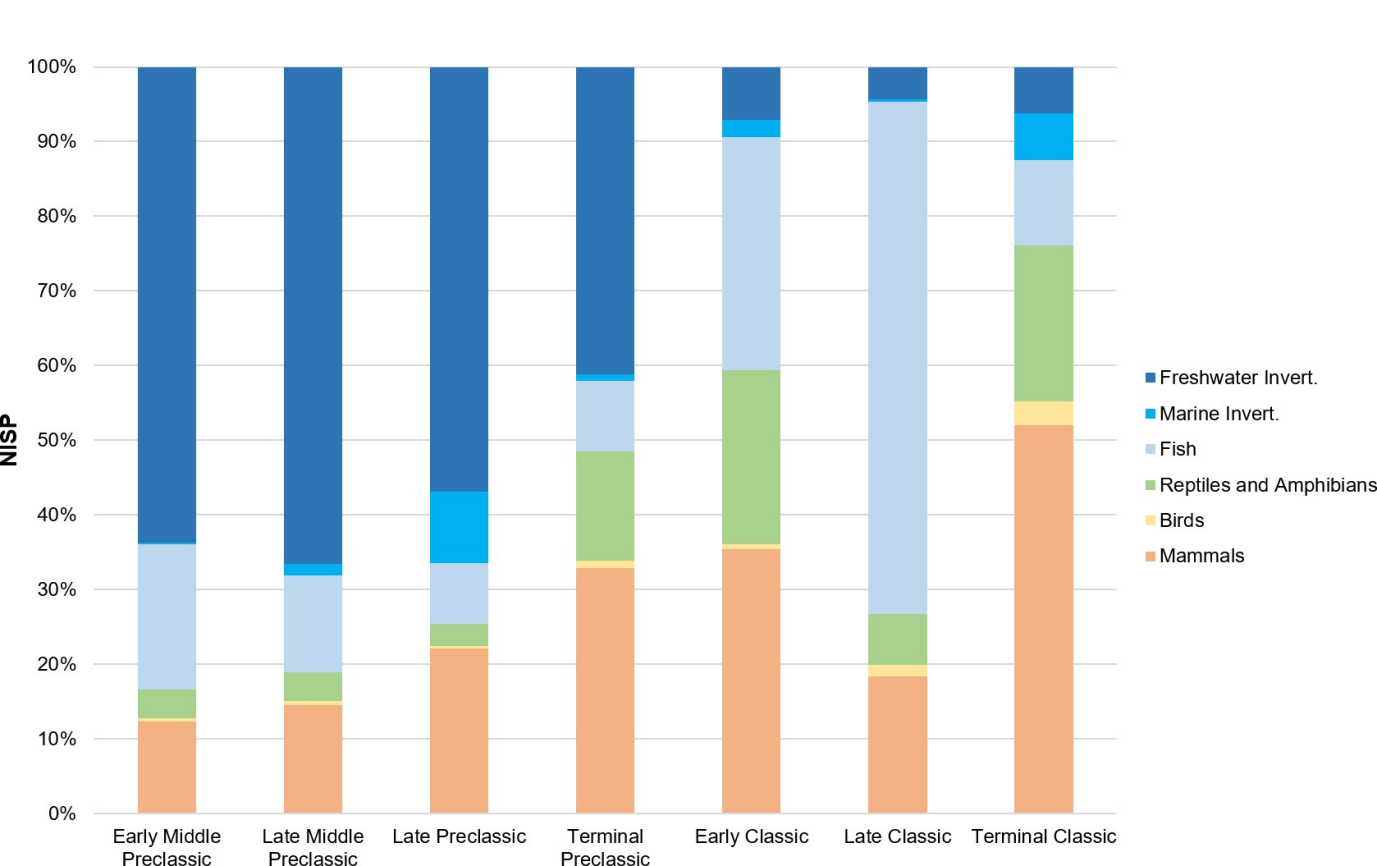
Number of identified specimens (NISP) at Ceibal over time.

**Fig 4:**
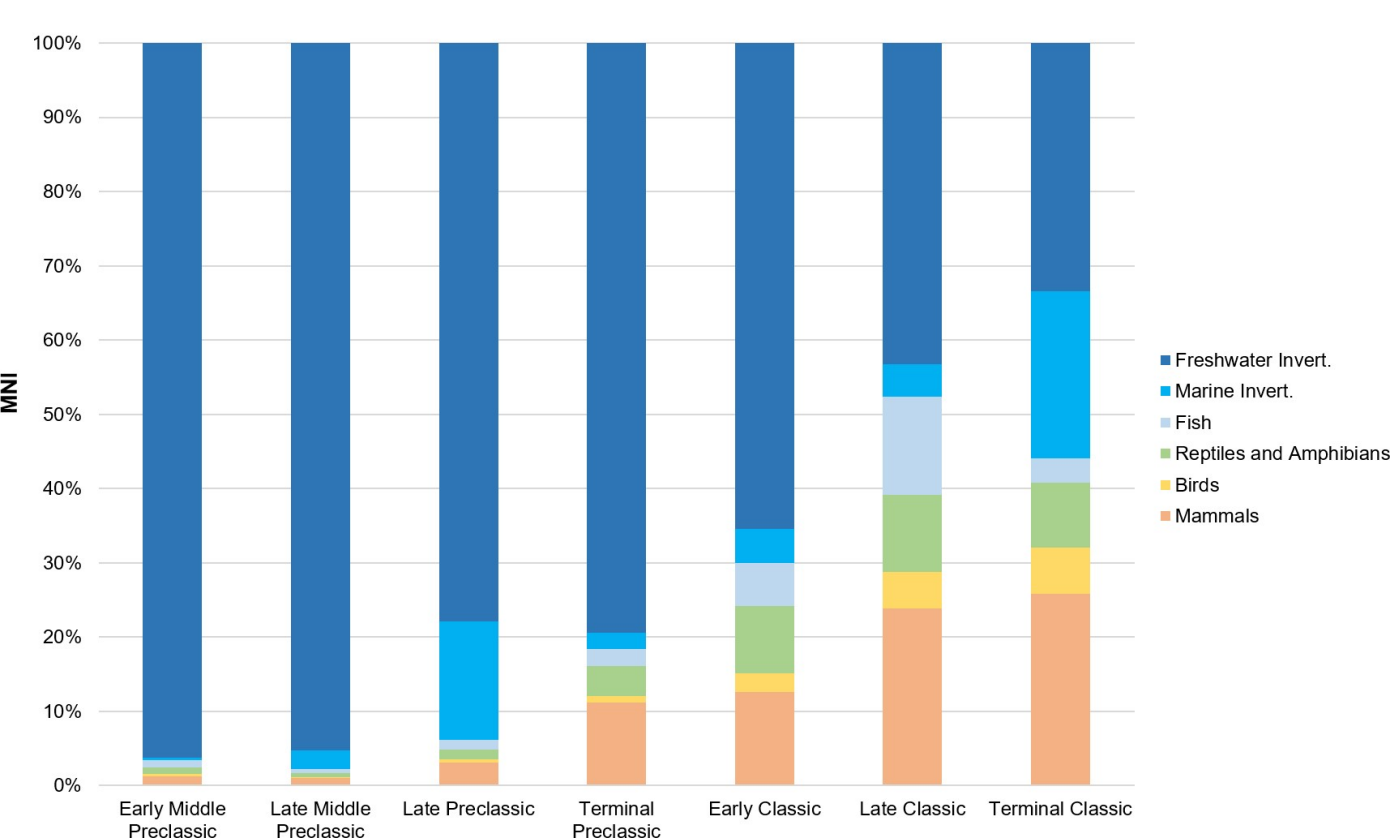
Minimum number of individuals (MNI) at Ceibal over time.

When comparing the major taxonomic categories at Ceibal and the outlying groups over time (Tables 2 and 3 and Figs 3 and 4), there is a noticeable shift between the proportion of vertebrates compared to invertebrates, occurring sometime between the Preclassic and Classic periods (c. AD 250). The Preclassic phases have a far greater proportion of invertebrates, which are mainly freshwater shellfish. The greatest number of these (NISP = 6579 at Ceibal, NISP = 1208 at Caobal; >90% total NISP of the period) occurs during the Middle Preclassic period. The Classic period inhabitants, especially during the Early and Late Classic, obtained more fish than the occupations of other periods. Reptiles and birds were proportionally similar in all periods according to their NISP counts, but their proportions double in the Classic period when referring to MNI, due both to an increase in reptile and bird specimens during the Classic period, as well as to the proportional decline in freshwater mollusks. Birds were always the lowest class (<4% of vertebrates by NISP each period). Mammals became the dominant taxa in the Terminal Classic (59% of vertebrates by NISP). The actual number of specimens (NISP) is in the thousands for most time periods. The lower number of specimens (NISP = 851) recovered from the Terminal Preclassic period compared to other periods may be due to the fact that there was a smaller population at the site during this period compared to other times, and also because some of the ceramics from this period, which is the principal means of dating excavations levels, overlap with that of the Late Preclassic. Thus, some of the levels designated as Late Preclassic may include Terminal Preclassic material. Patterns among MNI counts are similar to NISP counts, but they likely greatly underestimate the proportion of vertebrate fauna compared to invertebrates, the latter of which are likely accurately reflected. Evidence of the Postclassic period (1000–1200 AD) was only found on Platform 97 (S1 Table); since the majority of specimens (>60%) from this period were terrestrial snails and only one identifiable bone was found, this period will only be briefly discussed in relation to the other phases.

**Table 2:** The number of individual specimens (NISP) and minimum number of individuals (MNI) at Ceibal.

		Early Middle Preclassic (Real-Xe)		Late Middle Preclassic (Escoba-Mamom)		Late Preclassic (Cantutse-Chicanel)		Terminal Preclassic (Xate)		Early Classic (Junco-Tzakol)		Late Classic (Tepejilote-Tepeu)		Terminal Classic (Bayal)		Total	
Scientific Name	Common Name	NISP	MNI	NISP	MNI	NISP	MNI	NISP	MNI	NISP	MNI	NISP	MNI	NISP	MNI	NISP	MNI
*Didelphis virginiana*	Virginia opossum	- -	- -	1 (0.03)	1 (0.69)	- -	- -	- -	- -	- -	- -	32 (0.65)	3 (1.57)	2 (0.07)	1 (0.47)	35	5
cf. *Philander opossum*	gray four-eyed opossum	- -	- -	1 (0.03)	1 (0.69)	3 (0.34)	2 (2.13)	- -	- -	13 (0.40)	3 (3.23)	11 (0.22)	5 (2.62)	25 (0.88)	10 (4.72)	53	21
*Philander* or *Caluromys* sp.	four-eyed or woolly opossum	- -	- -	- -	- -	1 (0.11)	1 (1.06)	- -	- -	- -	- -	- -	- -	- -	- -	1	2
Didelphidae	opossums	4 (0.27)	2 (2.44)	2 (0.06)	1 (0.69)	2 (0.23)	2 (2.13)	- -	- -	- -	- -	8 (0.16)	2 (1.05)	2 (0.07)	2 (0.94)	18	9
Chiroptera	bats	- -		- -	- -	- -	- -	- -	- -	- -	- -	- -	- -	1 (0.04)	1 (0.47)	1	1
cf. *Alouatta pigra*	Guatemalan black howler monkey	- -		- -	- -	- -	- -	- -	- -	- -	- -	- -	- -	1 (0.04)	1 (0.47)	1	1
cf. *Ateles geoffroyi*	Geoffroy's spider monkey	- -		- -	- -	- -	- -	- -	- -	- -	- -	3 (0.06)	1 (0.52)	- -	- -	3	1
*Tamandua mexicana*	northern tamandua (anteater)	- -		- -	- -	- -	- -	- -	- -	- -	- -	- -	- -	1 (0.04)	1 (0.47)	1	1
*Dasypus novemcinctus*	nine-banded armadillo	47 [3] (0.20)	2 (2.44)	9 (0.29)	6 (4.14)	2 (0.23)	2 (2.13)	- -	- -	11 [6] (0.18)	4 (4.30)	6 [5] (0.10)	4 (2.09)	13 [10] (0.35)	7 (3.30)	35	25
*Sylvilagus* sp.	rabbits	1 (0.07)	1 (1.22)	8 (0.25)	3 (2.07)	- -	- -	- -	- -	1 (0.03)	1 (1.08)	- -	- -	5 (0.18)	4 (1.89)	15	9
*Orthogeomys hispidus*	hispid pocket gopher	- -	- -	- -	- -	2 (0.23)	1 (1.06)	1 (0.32)	1 (2.44)	2 (0.06)	1 (1.08)	- -	- -	4 (0.14)	2 (0.94)	9	5
Heteromyidae	pocket mice	- -	- -	- -	- -	- -	- -	- -	- -	- -	- -	- -	- -	1 (0.04)	1 (0.47)	1	1
cf. *Peromyscus* sp.	deer mice	- -	- -	- -	- -	- -	- -	4 (1.27)	1 (2.44)	- -	- -	- -	- -	- -	- -	4	1
Sigmodontinae	New World rodents	- -	- -	- -	- -	- -	- -	- -	- -	- -	- -	- -	- -	1 (0.04)	1 (0.47)	1	1
*Dasyprocta punctata*	Central American agouti	- -	- -	1 (0.03)	1 (0.69)	1 (0.11)	1 (1.06)	- -	- -	1 (0.03)	1 (1.08)	12 (0.24)	8 (4.19)	15 (0.53)	7 (3.30)	30	18
*Cuniculus paca*	lowland paca	3 (0.20)	3 (3.66)	3 (0.10)	2 (1.38)	2 (0.23)	2 (2.13)	1 (0.32)	1 (2.44)	1 (0.03)	1 (1.08)	- -	- -	9 (0.32)	3 (1.42)	19	12
Caviomorpha	agoutis and pacas	1 (0.07)	1 (1.22)	- -	- -	1 (0.11)	1 (1.06)	- -	- -	- -	- -	1 (0.02)	1 (0.52)	1 (0.04)	1 (0.47)	4	4
Rodentia, medium size	rodents (size of squirrel or gopher)	- -	- -	1 (0.03)	1 (0.69)	- -	- -	- -	- -	- -	- -	2 (0.04)	2 (1.05)	1 (0.04)	1 (0.47)	4	4
Rodentia, small size	rodents (size of rice rat or cotton mouse)	4 (0.27)	2 (2.44)	15 (0.48)	6 (4.14)	1 (0.11)	1 (1.06)	7 (2.22)	2 (4.88)	3 (0.09)	1 (1.08)	10 (0.20)	4 (2.09)	18 (0.63)	7 (3.30)	58	23
*Canis lupus familiaris*	domestic dog	54 (3.68)	8 (9.76)	363 (11.54)	24 (16.55)	44 (5.03)	14 (14.89)	6 (1.90)	4 (9.76)	652 (19.93)	5 (5.38)	48 (0.97)	10 (5.24)	47 (1.65)	9 (4.25)	1214	74
*Urocyon cinereoargenteus*	gray fox	2 (0.14)	1 (1.22)	3 (0.10)	2 (1.38)	1 (0.11)	1 (1.06)	- -	- -	1 (0.03)	1 (1.08)	4 (0.08)	4 (2.09)	6 (0.21)	2 (0.94)	17	11
Canidae	dogs and foxes	- -	- -	6 (0.19)	- -	- -	- -	- -	- -	- -	- -	- -	- -	2 (0.07)	1 (0.47)	8	1
*Procyon lotor*	raccoon	2 (0.14)	1 (1.22)	- -	- -	- -	- -	- -	- -	- -	- -	1 (0.02)	1 (0.52)	1 (0.04)	1 (0.47)	4	3
*Nasua narica*	white-nosed coati	- -	- -	- -	- -	- -	- -	- -	- -	1 (0.03)	1 (1.08)	11 (0.22)	2 (1.05)	- -	- -	12	3
*Potos flavus*	kinkajou	- -	- -	- -	- -	- -	- -	- -	- -	- -	- -	1 (0.02)	1 (0.52)	5 (0.18)	2 (0.94)	6	3
Procyonidae	raccoon, coati, or kinkajou	- -	- -	- -	- -	- -	- -	- -	- -	- -	- -	1 (0.02)	1 (0.52)	1 (0.04)	1 (0.47)	2	2
*Galictis vittata*	greater grison	- -	- -	- -	- -	- -	- -	- -	- -	- -	- -	1 (0.02)	1 (0.52)	- -	- -	1	1
cf. *Mustela frenata*	long-tailed weasel	1 (0.07)	1 (1.22)	- -	- -	- -	- -	- -	- -	- -	- -	- -	- -	- -	- -	1	1
*Leopardus* cf. *pardalis*	ocelot	- -	- -	- -	- -	- -	- -	- -	- -	- -	- -	2 (0.04)	2 (1.05)	- -	- -	2	2
*Leopardus* cf. *wiedii*	margay	- -	- -	- -	- -	- -	- -	- -	- -	- -	- -	- -	- -	2 (0.07)	1 (0.47)	2	1
*Leopardus* sp.	ocelots and margays	1 (0.07)	1 (1.22)	- -	- -	- -	- -	- -	- -	- -	- -	2 (0.04)	1 (0.52)	2 (0.07)	2 (0.94)	5	4
*Panthera onca*	jaguar	- -	- -	4 (0.13)	2 (1.38)	- -	- -	- -	- -	- -	- -	- -	- -	3 (0.11)	3 (1.42)	7	5
*Puma concolor*	puma	- -	- -	1 (0.03)	1 (0.69)	1 (0.11)	1 (1.06)	- -	- -	- -	- -	- -	- -	1 (0.04)	1 (0.47)	3	3
Felidae, small	jaguar, puma, ocelot, or margray	- -	- -	- -	- -	- -	- -	- -	- -	- -	- -	- -	- -	1 (0.04)	1 (0.47)	1	1
Felidae, large	jaguar or puma	- -	- -	2 (0.06)	1 (0.69)	2 (0.23)	1 (1.06)	- -	- -	2 (0.06)	1 (1.08)	- -	- -	10 (0.35)	1 (0.47)	16	4
Carnivora	carnivores	10 (0.68)	- -	15 (0.48)	- -	6 (0.69)	- -	2 (0.63)	- -	3 (0.09)	- -	10 (0.20)	- -	10 (0.35)	1 (0.47)	56	1
Carnivora, small size	small carnivores (size of weasel)	1 (0.07)	- -	1 (0.03)	- -	1 (0.11)	1 (1.06)	- -	- -	- -	- -	1 (0.02)	- -	2 (0.07)	- -	6	1
*Tapirella bairdii*	Baird's tapir	1 (0.07)	1 (1.22)	6 (0.19)	1 (0.69)	4 (0.46)	2 (2.13)	2 (0.63)	2 (4.88)	2 (0.06)	1 (1.08)	- -	- -	2 (0.07)	1 (0.47)	17	8
Tayassuidae	peccaries	2 (0.14)	2 (2.44)	2 (0.06)	1 (0.69)	1 (0.11)	1 (1.06)	5 (1.59)	4 (9.76)	12 (0.37)	4 (4.30)	9 (0.18)	8 (4.19)	14 (0.49)	6 (2.83)	45	26
*Mazama* sp.	brocket deer	- -	- -	1 (0.03)	1 (0.69)	2 (0.23)	2 (2.13)	1 (0.32)	1 (2.44)	6 (0.18)	2 (2.15)	3 (0.06)	2 (1.05)	9 (0.32)	3 (1.42)	22	11
*Odocoileus virginianus*	white-tailed deer	26 (1.77)	4 (4.88)	83 (2.64)	12 (8.28)	67 (7.66)	10 (10.64)	25 (7.94)	9 (21.95)	70 (2.14)	10 (10.75)	228 (4.60)	23 (12.04)	287 (10.09)	36 (16.98)	786	104
Cervidae	deer	5 (0.34)	1 (1.22)	8 (0.25)	- -	7 (0.80)	2 (2.13)	- -	- -	9 (0.27)	2 (2.15)	9 (0.18)	1 (0.52)	15 (0.53)	2 (0.94)	53	8
Artiodactyla	deer or peccary	1 (0.07)	- -	1 (0.03)	- -	- -	- -	- -	- -	3 (0.09)	- -	3 (0.06)	- -	3 (0.11)	- -	11	0
Mammalia	unidentified mammals	250 (17.04)	- -	559 (17.77)	- -	243 (27.77)	- -	69 (21.90)	- -	293 (8.95)	- -	327 (6.59)	- -	640 (22.51)	- -	2381	0
Mammalia, medium-large size	unidentified mammals (size of deer or jaguar)	91 (6.20)	- -	214 (6.80)	- -	164 (18.74)	- -	47 (14.92)	- -	153 (4.67)	- -	135 (2.72)	- -	449 (15.79)	- -	1253	0
Mammalia, medium-small size	unidentified mammals (size of dog or opossum)	35 (2.39)	- -	113 (3.59)	- -	16 (1.83)	- -	9 (2.86)	- -	43 (1.31)	- -	62 (1.25)	- -	73 (2.57)	- -	351	0
Mammalia, small size	unidentified mammals (size of bat or rat)	1 (0.07)	- -	14 (0.45)	- -	3 (0.34)	- -	- -	- -	- -	- -	10 (0.20)	- -	6 (0.21)	- -	34	0
**Total Mammals**		**499 (34.01)**	**31 (37.80)**	**1437 (45.68)**	**67 (46.21)**	**577 (65.94)**	**48 (51.06)**	**179 (56.83)**	**25 (60.98)**	**1277 (39.00)**	**39 (41.94)**	**952 (19.19)**	**87 (45.55)**	**1688 (59.37)**	**124 (58.49)**	**6609**	**421**
cf. *Colinus virginianus*	northern bobwhite	- -	- -	1 (0.03)	1 (0.69)	- -	- -	- -	- -	- -	- -	- -	- -	- -	- -	1	1
cf. *Penelope purpurascens*	crested guan	- -	- -	- -	- -	- -	- -	- -	- -	- -	- -	- -	- -	7 (0.25)	3 (1.42)	7	3
*Ortalis vetula*	plain chachalaca	- -	- -	- -	- -	- -	- -	- -	- -	- -	- -	- -	- -	1 (0.04)	1 (0.47)	1	1
*Meleagris gallopavo*	wild turkey	- -	- -	- -	- -	- -	- -	- -	- -	- -	- -	- -	- -	3 (0.11)	2 (0.94)	3	2
*Meleagris ocellata*	ocellated turkey	- -	- -	- -	- -	- -	- -	- -	- -	- -	- -	1 (0.02)	1 (0.52)	- -	- -	1	1
*Meleagris* sp.	turkeys	- -	- -	- -	- -	- -	- -	1 (0.32)	1 (2.44)	1 (0.03)	1 (1.08)	12 (0.24)	4 (2.09)	14 (0.49)	7 (3.30)	28	13
Galliformes	turkeys, quails, guans	- -	- -	- -	- -	- -	- -	- -	- -	1 (0.03)	1 (1.08)	1 (0.02)	1 (0.52)	9 (0.32)	- -	11	2
cf. *Aythya* sp.	ducks	1 (0.07)	1 (1.22)	- -	- -	- -	- -	- -	- -	- -	- -	- -	- -	- -	- -	1	1
cf. *Dendrocygna* sp.	whistling duck	- -	- -	1 (0.03)	1 (0.69)	- -	- -	- -	- -	- -	- -	1 (0.02)	1 (0.52)	- -	- -	2	2
*Cairina moschata*	muscovy duck	- -	- -	- -	- -	- -	- -	- -	- -	- -	- -	- -	- -	1 (0.04)	1 (0.47)	1	1
Anatidae	ducks and geese	1 (0.07)	- -	- -	- -	- -	- -	- -	- -	- -	- -	- -	- -	- -	- -	1	0
*Egretta* cf. *caerulea*	little blue heron	- -	- -	1 (0.03)	1 (0.69)	- -	- -	- -	- -	- -	- -	- -	- -	- -	- -	1	1
*Egretta* sp.	herons	- -	- -	- -	- -	- -	- -	- -	- -	- -	- -	- -	- -	4 (0.14)	2 (0.94)	4	2
*Nycticorax nycticorax*	black-crowned night heron	- -	- -	- -	- -	- -	- -	- -	- -	- -	- -	1 (0.02)	1 (0.52)	- -	- -	1	1
Ardeidae	herons	- -	- -	- -	- -	- -	- -	- -	- -	- -	- -	1 (0.02)	1 (0.52)	1 (0.04)	1 (0.47)	2	2
cf. *Anhinga anhinga*	anhinga (darter)	- -	- -	- -	- -	- -	- -	- -	- -	- -	- -	1 (0.02)	1 (0.52)	- -	- -	1	1
cf. Accipitridae	hawks	- -	- -	- -	- -	- -	- -	- -	- -	1 (0.03)	1 (1.08)	- -	- -	- -	- -	1	1
Falconidae	falcons	- -	- -	- -	- -	- -	- -	- -	- -	- -	- -	- -	- -	1 (0.04)	1 (0.47)	1	1
cf. Cathartidae	New World vultures	- -	- -	20 (0.64)	1 (0.69)	- -	- -	- -	- -	- -	- -	1 (0.02)	1 (0.52)	- -	- -	21	2
cf. *Aramus guarauna* (Gruiformes)	tails, trumpeters (limpkin?)	- -	- -	- -	- -	- -	- -	- -	- -	1 (0.03)	1 (1.08)	- -	- -	- -	- -	1	1
cf. *Tyto alba*	barn owl	1 (0.07)	1 (1.22)	- -	- -	- -	- -	- -	- -	- -	- -	- -	- -	- -	- -	1	1
cf. *Quiscalus mexicanus*	great-tailed grackle	- -	- -	1 (0.03)	1 (0.69)	- -	- -	- -	- -	- -	- -	- -	- -	- -	- -	1	1
Aves	unidentified birds	2 (0.14)	- -	17 (0.54)	1 (0.69)	3 (0.34)	2 (2.13)	4 (1.27)	1 (2.44)	14 (0.43)	- -	46 (0.93)	1 (0.52)	29 (1.02)	4 (1.89)	115	9
Aves, medium-large size	unidentified birds (size of a turkey)	6 (0.41)	2 (2.44)	1 (0.03)	- -	1 (0.11)	1 (1.06)	- -	- -	- -	- -	5 (0.10)	3 (1.57)	16 (0.56)	1 (0.47)	29	7
Aves, medium-small size	unidentified birds (size of a duck or hawk)	5 (0.34)	1 (1.22)	10 (0.32)	1 (0.69)	3 (0.34)	2 (2.13)	- -	- -	7 (0.21)	2 (2.15)	8 (0.16)	1 (0.52)	14 (0.49)	4 (1.89)	47	11
Aves, small size	unidentified birds (size of a quail or dove)	1 (0.07)	1 (1.22)	2 (0.06)	- -	1 (0.11)	1 (1.06)	- -	- -	2 (0.06)	2 (2.15)	2 (0.04)	2 (1.05)	4 (0.14)	3 (1.42)	12	9
**Total Birds**		**17 (1.16)**	**6 (7.32)**	**54 (1.72)**	**7 (4.83)**	**8 (0.91)**	**6 (6.38)**	**5 (1.59)**	**2 (4.88)**	**27 (0.82)**	**8 (8.60)**	**80 (1.61)**	**18 (9.42)**	**104 (3.66)**	**30 (14.15)**	**295**	**77**
*Chelydra rossignonii*	snapping turtle	1 [1] (0.07)	1 (1.22)	5 [5] (0.16)	1 (0.69)	- -	- -	- -	- -	- -	- -	- -	- -	- -	- -	6 (6)	2
*Trachemys venusta*	Mesoamerican slider	52 [4] (3.54)	3 (3.66)	124 [9] (3.94)	**7 (4.83)**	29 [4] (3.31)	4 (4.26)	13 [2] (4.13)	2 (4.88)	30 [5] (0.92)	3 (3.23)	28 [14] (0.56)	7 (3.66)	275 [9] (9.67)	8 (3.77)	551 (47)	34
*Rhinoclemmys* or *Trachemys*	wood turtle or slider	- -	- -	1 (0.03)	1 (0.69)	- -	- -	- -	- -	- -	- -	- -	- -	- -	- -	1	1
*Dermatemys mawii*	Central American river turtle	12 [3] (0.82)	3 (3.66)	33 [5] (1.05)	2 (1.38)	6 [4] (0.69)	3 (3.19)	57 [3] (18.10)	3 (7.32)	582 [22] (17.78)	8 (8.60)	95 [13] (1.92)	7 (3.66)	206 [15] (7.25)	10 (4.72)	991 (65)	36
*Dermatemys* or *Trachemys*	river turtle or slider	- -	- -	- -	- -	5 (0.57)	- -	- -	- -	9 (0.27)	2 (2.15)	1 (0.02)	- -	14 (0.49)	1 (0.47)	29	3
*Staurotypus triporcatus*	Mexican musk turtle	28 [6] (1.91)	6 (7.32)	20 [6] (0.64)	3 (2.07)	1 [1] (0.11)	1 (1.06)	- -	- -	2 [2] (0.06)	1 (1.08)	7 [2] (0.14)	2 (1.05)	8 [3] (0.28)	2 (0.94)	66 (20)	15
Kinosternidae/Staurotypidae	mud or musk turtle	11 [3] (0.75)	3 (3.66)	35 [8] (1.11)	7 (4.83)	5 [3] (0.57)	3 (3.19)	- -	- -	14 [5] (0.43)	2 (2.15)	7 [4] (0.14)	4 (2.09)	2 [2] (0.07)	2 (0.94)	74 (25)	21
*Chelydra* or *Staurotypus*	snapping or Mexican musk turtle	- -	- -	- -	- -	- -	- -	- -	- -	- -	- -	- -	- -	1 (0.04)	- -	1	0
Testudines	unidentified turtles	31 (2.11)	1 (1.22)	86 (2.73)	1 (0.69)	11 (1.26)	- -	5 (1.59)	2 (4.88)	149 (4.55)	1 (1.08)	50 (1.01)	2 (1.05)	74 (2.60)	3 (1.42)	406	10
Testudines, medium-large size	unidentified turtle (size of river turtle)	11 (0.75)	- -	34 (0.82)	1 (0.69)	11 (1.26)	3 (3.19)	3 (0.95)	- -	28 (0.86)	- -	43 (0.87)	1 (0.52)	79 (2.78)	3 (1.42)	209	8
Testudines, small size	unidentified turtle (size of small mud turtle)	7 (0.48)		8 (0.25)	- -	- -	- -	1 (0.32)	1 (2.44)	2 (0.06)	- -	8 (0.16)	1 (0.52)	1 (0.04)	- -	27	2
Iguanidae	iguanas	- -	- -	1 (0.03)	1 (0.69)	4 (0.46)	2 (2.13)	- -	- -	3 (0.09)	2 (2.15)	3 (0.06)	1 (0.52)	2 (0.07)	2 (0.94)	13	8
cf. Teiidae	whiptails	- -	- -	1 (0.03)	1 (0.69)	- -	- -	- -	- -	- -	- -	- -	- -	- -	- -	1	1
Lacertilia, small	unidentified small lizard	1 (0.07)	1 (1.22)	4 (0.13)	3 (2.07)	2 (0.23)	1 (1.06)	- -	- -	2 (0.06)	1 (1.08)	1 (0.02)	1 (0.52)	2 (0.07)	2 (0.94)	12	9
cf. Viperidae	vipers	- -	- -	1 (0.03)	1 (0.69)	- -	- -	- -	- -	- -	- -	- -	- -	- -	- -	1	1
Serpentes	snakes	- -	- -	3 (0.10)	1 (0.69)	1 (0.11)	1 (1.06)	- -	- -	3 (0.09)	3 (3.23)	1 (0.02)	1 (0.52)	4 (0.14)	4 (1.89)	12	10
Crocodylidae	crocodile or caiman	1 (0.07)	1 (1.22)	- -	- -	1 (0.11)	1 (1.06)	- -	- -	11 (0.34)	2 (2.15)	28 (0.56)	3 (1.57)	6 (0.21)	2 (0.94)	47	9
Reptilia	unidentified reptiles	- -	- -	- -	- -	1 (0.11)	- -	- -	- -	- -	- -	3 (0.06)	1 (0.52)	1 (0.04)	1 (0.47)	5	2
Reptilia, large	unidentified reptiles (size of a crocodile or large iguana)	- -	- -	- -	- -	- -	- -	- -	- -	- -	- -	2 (0.04)	2 (1.05)	- -	- -	2	2
Reptilia, small	unidentified reptiles (size of a gecko or small snake)	- -	- -	10 (0.32)	1 (0.69)	- -	- -	- -	- -	- -	- -	- -	- -	1 (0.04)	- -	11	1
**Total Reptiles**		**155 (10.57)**	**19 (23.17)**	**366 (11.63)**	**31 (21.38)**	**77 (8.80)**	**19 (20.21)**	**79 (25.08)**	**8 (19.51)**	**835 (25.50)**	**25 (26.88)**	**277 (5.58)**	**33 (17.28)**	**676 (23.78)**	**40 (18.87)**	**2465**	**175**
Dermophiidae	common caecilian	- -	- -	- -	- -	- -	- -	- -	- -	- -	- -	74 (1.49)	1 (0.52)	- -	- -	74	1
cf. *Rhinella marina*	cane toad	- -	- -	- -	- -	- -	- -	- -	- -	- -	- -	2 (0.04)	1 (0.52)	1 (0.04)	1 (0.47)	3	2
Anura	frogs and toads	4 (0.27)	2 (2.44)	8 (0.25)	5 (3.45)	2 (0.23)	2 (2.13)	1 (0.32)	1 (2.44)	6 (0.18)	3 (3.23)	6 (0.12)	3 (1.57)	1 (0.04)	1 (0.47)	28	17
**Total Amphibians**		4 (0.27)	2 (2.44)	8 (0.25)	5 (3.45)	2 (0.23)	2 (2.13)	1 (0.32)	1 (2.44)	6 (0.18)	3 (3.23)	82 (1.65)	5 (2.62)	2 (0.07)	2 (0.94)	105	20
*Atractosteus tropicus*	tropical gar	102 (6.95)	5 (6.10)	102 (3.24)	4 (2.76)	19 (2.17)	7 (7.45)	4 (1.27)	2 (4.88)	161 (4.92)	4 (4.30)	810 (16.33)	5 (2.62)	47 (1.65)	4 (1.89)	1245	31
cf. *Potamarius* sp. (large Ariidae)	hardhead or sea catfish	1 (0.07)	1 (1.22)	2 (0.06)	2 (1.38)	1 (0.11)	1 (1.06)	- -	- -	3 (0.09)	3 (3.23)	- -	- -	- -	- -	7	7
*Cathorops* sp.	catfish	5 (0.34)	4 (4.88)	6 (0.19)	4 (2.76)	- -	- -	- -	- -	3 (0.09)	2 (2.15)	16 (0.32)	10 (5.24)	1 (0.04)	1 (0.47)	31	21
Siluriformes	catfish	84 (5.73)	9 (10.98)	104 (3.31)	14 (9.66)	17 (1.94)	9 (9.57)	1 (0.32)	1 (2.44)	29 (0.89)	2 (2.15)	194 (3.91)	24 (12.57)	15 (0.53)	5 (2.36)	444	64
Cichlidae	cichlids	8 (0.55)	3 (3.66)	27 (0.86)	9 (6.21)	6 (0.69)	2 (2.13)	- -	- -	9 (0.27)	4 (4.30)	20 (0.40)	5 (2.62)	5 (0.18)	4 (1.89)	75	27
*Centropomus* sp.	snook	- -	- -	2 (0.06)	1 (0.69)	- -	- -	- -	- -	12 (0.37)	1 (1.08)	1 (0.02)	1 (0.52)	- -	- -	15	3
*Aplodinotus grunniens*	freshwater drum	2 (0.14)	2 (2.44)	2 (0.06)	1 (0.69)	- -	- -	2 (0.63)	2 (4.88)	5 (0.15)	2 (2.15)	8 (0.16)	3 (1.57)	2 (0.07)	1 (0.47)	21	11
Actinopterygii	unidentified bony fish	590 (40.22)	- -	1036 (32.93)	- -	168 (19.20)	- -	44 (13.97)	- -	907 (27.70)	- -	2520 (50.81)	- -	302 (10.62)	- -	5567	n/a
**Total Bony Fish**		**792 (53.99)**	**24 (29.27)**	**1281 (40.72)**	**35 (24.14)**	**211 (24.11)**	**19 (20.21)**	**51 (16.19)**	**5 (12.20)**	**1129 (34.48)**	**18 (19.35)**	**3569 (71.96)**	**48 (25.13)**	**372 (13.08)**	**15 (7.08)**	**7405**	**164**
*Carcharhinus* cf. *leucas*	bull shark	- -	- -	- -	- -	- -	- -	- -	- -	- -	- -	- -	- -	1 (0.04)	1 (0.47)	1	1
**Total Cartilaginous Fish**		- -	- -	- -	- -	- -	- -	- -	- -	- -	- -	- -	- -	1 (0.04)	1 (0.47)	1	1
**Total Identified Vertebrates**		**1467 (100)**	**82 (100)**	**3146 (100)**	**145 (100)**	**875 (100)**	**94 (100)**	**315 (100)**	**41 (100)**	**3274 (100)**	**93 (100)**	**4960 (100)**	**191 (100)**	**2843 (100)**	**212 (100)**	**16880**	**858**
**Total Unidentified Vertebrates**^a^		**333**	**—**	**902**	**—**	**240**	**—**	**60**	**—**	**546**	**—**	**894**	**—**	**516**	**—**	**3491**	**n/a**
*Spondylus* sp.	thorny oysters	1 (0.04)	1 (0.04)	15 [2] (0.03)	2 (0.03)	- -	- -	2 (0.37)	2 (0.41)	- -	- -	- -	- -	- -	- -	5	5
*Crassostrea* sp.	common/true oyster	- -	- -	- -	- -	- -	- -	- -	- -	- -	- -	- -	- -	1 (0.03)	1 (0.03)	1	1
*Noetia ponderosa*	ponderous ark clam	- -	- -	- -	- -	- -	- -	2 (0.37)	2 (0.41)	- -	- -	- -	- -	- -	- -	2	2
Arcidae, small	small ark clam (<1cm length)	- -	- -	1 (0.01)	1 (0.01)	- -	- -	- -	- -	- -	- -	- -	- -	- -	- -	1	1
*Donax* cf. *denticulatus*	common caribbean donax/bean clam	- -	- -	1 (0.01)	1 (0.01)	- -	- -	- -	- -	- -	- -	- -	- -	- -	- -	1	1
Veneridae	venus clams	- -	- -	- -	- -	- -	- -	- -	- -	1 (0.22)	1 (0.31)	- -	- -	- -	- -	1	1
Bivalvia, marine, small	small marine bivalve (<1cm length)	- -	- -	1 (0.01)	1 (0.01)	- -	- -	- -	- -	- -	- -	- -	- -	- -	- -	1	1
Dentaliidae (cf. *Graptacme ebeora*)	tusk shell	4 (0.14)	4 (0.16)	16 (0.22)	16 (0.24)	1 (0.05)	1 (0.06)	- -	- -	1 (0.22)	1 (0.31)	- -	- -	1 (0.03)	1 (0.03)	23	23
*Diodora cayenensis* or *aspera*	limpet	- -	- -	- -	- -	1 (0.05)	1 (0.06)	- -	- -	- -	- -	- -	- -	- -	- -	1	1
*Nerita versicolor*	four-toothed nerite	- -	- -	- -	- -	- -	- -	- -	- -	- -	- -	- -	- -	1 (0.03)	1 (0.03)	1	1
*Conasprella* cf. *delessertii*	Sozon's cone snail	- -	- -	1 (0.01)	1 (0.01)	- -	- -	- -	- -	- -	- -	- -	- -	- -	- -	1	1
Conidae	cone snails	- -	- -	- -	- -	- -	- -	- -	- -	- -	- -	1 (0.12)	1 (0.13)	- -	- -	1	1
Olivinae	olive snails	- -	- -	22 (0.30)	21 (0.31)	4 (0.22)	4 (0.26)	1 (0.19)	1 (0.21)	2 (0.44)	2 (0.62)	5 (0.58)	5 (0.64)	88 (2.55)	57 (1.72)	122	90
*Olivella* cf. *nivea*	snowy dwarf olive	- -	- -	1 (0.01)	1 (0.01)	- -	- -	- -	- -	- -	- -	- -	- -	- -	- -	1	1
*Olivella* sp.	dwarf olive	- -	- -	- -	- -	- -	- -	- -	- -	- -	- -	1 (0.12)	1 (0.13)	- -	- -	1	1
*Cassis* sp. (*madagascariensis*?)	queen helmet	- -	- -	- -	- -	- -	- -	- -	- -	- -	- -	- -	- -	1 (0.03)	1 (0.03)	1	1
*Prunum apicinum*	Atlantic marginella	2 (0.07)	2 (0.08)	80 (1.10)	80 (1.18)	236 (12.72)	233 (14.93)	- -	- -	2 (0.44)	2 (0.62)	- -	- -	1 (0.03)	1 (0.03)	321	318
*Prunum* cf. *labiatum*	royal marginella	- -	- -	1 (0.01)	1 (0.01)	- -	- -	- -	- -	- -	- -	- -	- -	- -	- -	1	1
*Prunum* cf. *amabile* or *labiatum*	Roosevelt's marginella or royal marginella	- -	- -	4 (0.06)	3 (0.04)	1 (0.05)	1 (0.06)	- -	- -	- -	- -	- -	- -	- -	- -	5	4
*Prunum* cf. *guttatum*	white-spotted marginella	- -	- -	1 (0.01)	1 (0.01)	- -	- -	- -	- -	- -	- -	- -	- -	- -	- -	1	1
*Prunum* sp.	marginella snails (not apicinum)	- -	- -	2 (0.03)	2 (0.03)	- -	- -	- -	- -	- -	- -	- -	- -	- -	- -	2	2
*Turbinella angulata*	West Indian chank snail	- -	- -	4 (0.06)	4 (0.06)	- -	- -	- -	- -	1 (0.22)	1 (0.31)	- -	- -	1 (0.03)	1 (0.03)	6	6
*Lobatus gigas*	queen conch	- -	- -	2 (0.03)	2 (0.03)	2 (0.11)	1 (0.06)	- -	- -	- -	- -	1 (0.12)	1 (0.13)	- -	- -	5	4
Strombidae	conch snails	- -	- -	1 (0.01)	1 (0.01)	- -	- -	- -	- -	- -	- -	- -	- -	1 (0.03)	1 (0.03)	2	2
*Columbella mercatoria*	dove snail	- -	- -	1 (0.01)	1 (0.01)	- -	- -	- -	- -	- -	- -	- -	- -	35 (1.02)	35 (1.06)	36	36
Gastropoda, marine	unidentified marine snails	2 (0.07)	2 (0.08)	6 (0.08)	6 (0.09)	5 (0.27)	5 (0.32)	- -	- -	5 (1.10)	4 (1.24)	2 (0.23)	2 (0.26)	4 (0.12)	4 (0.12)	24	23
Gastropoda, large marine	unidentified large marine snails	- -	- -	12 (0.17)	11 (0.16)	- -	- -	- -	- -	1 (0.22)	1 (0.31)	5 (0.58)	3 (0.39)	1 (0.03)	3 (0.09)	21	18
Mollusca, marine	unidentified marine mollusks	- -	- -	1 (0.01)	1 (0.01)	- -	- -	- -	- -	72 (15.82)	2 (0.62)	2 (0.23)	2 (0.26)	50 (1.45)	1 (0.03)	125	6
**Total Marine Mollusks**		**9 (0.33)**	**9 (0.36)**	**160 (2.21)**	**157 (2.33)**	**250 (13.47)**	**246 (15.76)**	**5 (0.93)**	**5 (1.03)**	**85 (18.68)**	**14 (4.33)**	**17 (1.96)**	**15 (1.93)**	**187 (5.42)**	**107 (3.24)**	**713**	**553**
cf. *Corbicula fluminea*^b^	Asian clam	- -	- -	- -	- -	- -	- -	- -	- -	- -	- -	- -	- -	1 (0.03)	1 (0.03)	1	1
Unionidae	river mussel	490 (17.75)	204 (8.26)	802 (11.06)	322 (4.77)	593 (31.95)	304 (19.47)	93 (17.35)	46 (9.45)	96 (21.10)	44 (13.62)	132 (15.22)	63 (8.10)	90 (2.61)	49 (1.48)	2296	1032
*Pachychilus* cf. *glaphyrus*	jute snail	84 (3.04)	84 (3.40)	17 (0.23)	17 (0.25)	51 (2.75)	51 (3.27)	13 (2.43)	13 (2.67)	20 (4.40)	20 (6.19)	10 (1.15)	10 (1.29)	34 (0.99)	34 (1.03)	229	229
*Pachychilus* cf. *indiorum*	jute snail	3 (0.11)	3 (0.12)	5 (0.07)	5 (0.07)	10 (0.54)	10 (0.64)	11 (2.05)	11 (2.26)	- -	- -	3 (0.35)	3 (0.39)	1 (0.03)	1 (0.03)	33	33
*Pachychilus* sp.	jute snails	- -	- -	3 (0.04)	3 (0.04)	9 (0.48)	9 (0.58)	1 (0.19)	1 (0.21)	8 (1.76)	8 (2.48)	1 (0.12)	1 (0.13)	4 (0.12)	4 (0.12)	26	26
*Pomacea flagellata*	Central American apple snail	2017 (73.05)	2017 (81.63)	5752 (79.29)	5752 (85.19)	824 (44.40)	824 (52.79)	106 (19.78)	106 (21.77)	131 (28.79)	131 (40.56)	81 (9.34)	81 (10.41)	72 (2.09)	72 (2.18)	8983	8983
**Total Freshwater Mollusks**		**2594 (93.95)**	**2308 (93.40)**	**6579 (90.69)**	**6099 (90.33)**	**1487 (80.12)**	**1198 (76.75)**	**224 (41.79)**	**177 (36.34)**	**255 (56.04)**	**203 (62.85)**	**227 (26.18)**	**158 (20.31)**	**202 (5.86)**	**161 (4.87)**	**11568**	**10304**
*Helicina cf*. *amoena*	angled dome snail	1 (0.04)	1 (0.04)	4 (0.06)	4 (0.06)	- -	- -	5 (0.93)	5 (1.03)	2 (0.44)	2 (0.62)	- -	- -	15 (0.44)	15 (0.45)	27	27
Helicinidae, small	smaill dome snails	- -	- -	- -	- -	- -	- -	1 (0.19)	1 (0.21)	2 (0.44)	2 (0.62)	101 (11.65)	101 (12.98)	2136 (61.95)	2136 (64.63)	2240	2240
*Neocyclotus dysoni*	common crater snail	14 (0.51)	14 (0.57)	46 (0.63)	46 (0.68)	13 (0.70)	13 (0.83)	19 (3.54)	19 (3.90)	27 (5.93)	27 (8.36)	90 (10.38)	90 (11.57)	381 (11.05)	381 (11.53)	590	590
Neocyclotidae, small	small crater snails	1 (0.04)	1 (0.04)	- -	- -	1 (0.05)	1 (0.06)	- -	- -	1 (0.22)	1 (0.31)	13 (1.50)	13 (1.67)	229 (6.64)	229 (6.93)	245	245
Xanthonichidae (cf. *Trichodiscina* sp.?)	button snails	- -	- -	- -	- -	- -	- -	- -	- -	- -	- -	2 (0.23)	2 (0.26)	10 (0.29)	10 (0.30)	12	12
*Orthalicus princeps*	princess cone snail	5 (0.18)	5 (0.20)	6 (0.08)	6 (0.09)	23 (1.24)	23 (1.47)	6 (1.12)	6 (1.23)	6 (1.32)	6 (1.86)	43 (4.96)	43 (5.53)	77 (2.23)	77 (2.33)	166	166
*Bulimulus* sp.	terrestrial cone snails	85 (3.08)	85 (3.44)	333 (4.59)	333 (4.93)	59 (3.18)	59 (3.78)	56 (10.45)	56 (11.50)	36 (7.91)	36 (11.15)	265 (30.57)	265 (34.06)	17 (0.49)	17 (0.51)	851	851
*Succinea* sp.	amber snails	- -	- -	2 (0.03)	2 (0.03)	- -	- -	- -	- -	- -	- -	- -	- -	- -	- -	2	2
*Orthalicidae*, small	small terrestrial cone snails	2 (0.07)	2 (0.08)	- -	- -	- -	- -	2 (0.37)	2 (0.41)	- -	- -	4 (0.46)	4 (0.51)	6 (0.17)	6 (0.18)	14	14
*Euglandina* sp.	wolfsnail or marauder snail	15 (0.54)	15 (0.61)	33 (0.45)	33 (0.49)	10 (0.54)	10 (0.64)	5 (0.93)	5 (1.03)	17 (3.74)	17 (5.26)	30 (3.46)	30 (3.86)	26 (0.75)	26 (0.79)	136	136
*Volutaxis* or *Rectaxis* sp.	barb or splinter snails	5 (0.18)	5 (0.20)	25 (0.34)	25 (0.37)	- -	- -	37 (6.90)	37 (7.60)	3 (0.66)	3 (0.93)	11 (1.27)	11 (1.41)	29 (0.84)	29 (0.88)	110	110
cf. Spiraxidae	marauder or oval snail	2 (0.07)	2 (0.08)	- -	- -	1 (0.05)	1 (0.06)	- -	- -	- -	- -	2 (0.23)	2 (0.26)	- -	- -	5	5
Gastropoda, similar to Physidae	sinistral snail, possibly bladder snail	3 (0.11)	3 (0.12)	14 (0.19)	14 (0.21)	2 (0.11)	2 (0.13)	152 (28.36)	152 (31.21)	1 (0.22)	1 (0.31)	- -	- -	4 (0.12)	4 (0.12)	176	176
Gastropoda, terrestrial	unidentified terrestrial snails	21 (0.76)	21 (0.85)	33 (0.45)	33 (0.49)	8 (0.43)	8 (0.51)	22 (4.10)	22 (4.52)	11 (2.42)	11 (3.41)	43 (4.96)	43 (5.53)	106 (3.07)	106 (3.21)	244	244
**Total Terrestrial Snails**		**154 (5.58)**	**154 (6.23)**	**496 (6.84)**	**496 (7.35)**	**117 (6.30)**	**117 (7.50)**	**305 (56.90)**	**305 (62.63)**	**106 (23.30)**	**106 (32.82)**	**604 (69.67)**	**604 (77.63)**	**3036 (88.05)**	**3036 (91.86)**	**4818**	**4818**
Gastropoda	unidentified snails	4 (0.14)	- -	19 (0.26)	- -	2 (0.11)	- -	2 (0.37)	- -	9 (1.98)	- -	18 (2.08)	- -	8 (0.23)	- -	62	n/a
**Total Mollusks**		**2761 (100)**	**2471 (100)**	**7254 (100)**	**6752 (100)**	**1856 (100)**	**1561 (100)**	**536 (100)**	**487 (100)**	**455 (100)**	**323 (100)**	**866 (99.88)**	**777 (99.87)**	**3433 (99.56)**	**3304 (99.97)**	**17161**	**15675**
*Eucidaris* sp.	sea urchins	- -	- -	- -	- -	- -	- -	- -	- -	- -	- -	1 (0.12)	1 (0.13)	15 (0.44)	1 (0.03)	16	2
**Total Echinodermata**		**—**	**—**	**—**	**—**	**—**	**—**	**—**	**—**	**—**	**—**	**1 (0.12)**	**1 (0.13)**	**15 (0.44)**	**1 (0.03)**	**16**	**2**
**Total Invertebrates**		**2761 (100)**	**2471 (100)**	**7254 (100)**	**6752 (100)**	**1856 (100)**	**1561 (100)**	**536 (100)**	**487 (100)**	**455 (100)**	**323 (100)**	**867 (100)**	**778 (100)**	**3448 (100)**	**3305 (100)**	**17177**	**15677**
**TOTAL**		**4228 (12.42)**	**2553 (15.44)**	**7254 (30.54)**	**6897 (41.71)**	**2731 (8.02)**	**1655 (10.01)**	**851 (2.50)**	**528 (3.19)**	**3729 (10.95)**	**416 (2.52)**	**5827 (17.11)**	**969 (5.86)**	**6291 (18.47)**	**3517 (21.27)**	**34057**	**16535**

In most cases, NISP represents the minimum number of elements in a context. Fragments of armadillo and turtle shells have two counts, an overall total NISP for all fragments and a lower number in brackets denoting the estimated number of total carapaces. Percentages are based on either total number of vertebrates or invertebrates per period. The final total percentages are based on the total NISP and MNI counts for all periods.

^a^ Final total value does not include vertebrates that cannot be placed in a class, since these are often small fragments.

^b^ *Corbicula fluminea* is a modern intrusive bivalve; it may have been transported from the river and deposited in the topsoil by a bird or other animal.

**Table 3:** The number of individual specimens (NISP) and minimum number of individuals (MNI) at Caobal.

		Early Middle Preclassic (Real-Xe)		Late Middle Preclassic (Escoba-Mamom)		Late Preclassic (Cantutse-Chicanel)		Terminal Preclassic (Xate)		Early Classic (Junco-Tzakol)		Late Classic (Tepejilote-Tepeu)		Terminal Classic (Bayal)		Total	
Scientific Name	Common Name	NISP	MNI	NISP	MNI	NISP	MNI	NISP	MNI	NISP	MNI	NISP	MNI	NISP	MNI	NISP	MNI
*Sylvilagus* sp.	rabbits	- -	- -	3 (4.76)	1 (12.50)	- -	- -	- -	- -	- -	- -	- -	- -	- -	- -	3	1
*Cuniculus paca*	lowland paca	- -	- -	1 (1.59)	1 (12.50)	- -	- -	- -	- -	- -	- -	- -	- -	- -	- -	1	1
*Canis lupus familiaris*	domestic dog	- -	- -	1 (1.59)	1 (12.50)	- -	- -	- -	- -	- -	- -	- -	- -	- -	- -	1	1
*Odocoileus virginianus*	white-tailed deer	- -	- -	4 (6.35)	1 (12.50)	- -	- -	- -	- -	2 (10.00)	1 (33.33)	- -	- -	- -	- -	6	2
Mammalia	unidentified mammals	- -	- -	24 (38.10)	- -	19 (90.48)	- -	- -	- -	8 (40.00)	- -	- -	- -	- -	- -	51	- -
Mammalia, medium-large size	unidentified mammals (size of deer or jaguar)	- -	- -	- -	- -	2 (9.52)	1 (100)	- -	- -	2 (10.00)	- -	1 (100.00)	2 (100.00)	3 (100.00)	2 (100.00)	8	4
Mammalia, medium-small size	unidentified mammals (size of dog or opossum)	- -	- -	1 (1.59)	- -	- -	- -	- -	- -	- -	- -	- -	- -	- -	- -	1	- -
**Total Mammals**		**0**	**0**	**34 (53.97)**	**4 (50.00)**	**21 (100)**	**1 (100)**	**0**	**0**	**12 (60.00)**	**1 (33.33)**	**1 (100.00)**	**1 (100.00)**	**3 (100.00)**	**2 (100.00)**	**71**	**9**
Aves	unidentified birds	- -	- -	4 (6.35)	1 (12.50)	- -	- -	- -	- -	- -	- -	- -	- -	- -	- -	4	1
**Total Birds**		**0**	**0**	**4 (6.35)**	**1 (12.50)**	**0**	**0**	**0**	**0**	**0**	**0**	**0**	**0**	**0**	**0**	**4**	**1**
*Trachemys venusta*	Mesoamerican slider	- -	- -	22 [1] (34.92)	1 (12.50)	- -	- -	- -	- -	- -	- -	- -	- -	- -	- -	22	1
Testudines	unidentified turtles	- -	- -	- -	- -	- -	- -	- -	- -	1 (5.00)	- -	- -	- -	- -	- -	1	- -
Testudines, medium-large size	unidentified turtle (size of river turtle)	- -	- -	- -	- -	- -	- -	- -	- -	6 (30.00)	1 (33.33)	- -	- -	- -	- -	6	1
**Total Reptiles**		**0**	**0**	**22 (34.92)**	**1 (12.50)**	**0**	**0**	**0**	**0**	**7 (35.00)**	**1 (33.33)**	**0**	**0**	**0**	**0**	**29**	**2**
*Atractosteus tropicus*	tropical gar	- -	- -	1 (1.59)	1 (12.50)	- -	- -	- -	- -	- -	- -	- -	- -	- -	- -	1	1
Siluriformes	catfish	- -	- -	1 (1.59)	1 (12.50)	- -	- -	- -	- -	- -	- -	- -	- -	- -	- -	1	1
Actinopterygii	unidentified bony fish	- -	- -	1 (1.59)	- -	- -	- -	- -	- -	1 (5.00)	1 (33.33)	- -	- -	- -	- -	2	1
**Total Bony Fish**		**0**	**0**	**3 (4.76)**	**2 (25.00)**	**0**	**0**	**0**	**0**	**1 (5.00)**	**1 (33.33)**	**0**	**0**	**0**	**0**	**4**	**3**
**Total Identified Vertebrates**		**0**	**0**	**63 (100)**	**8 (100)**	**21 (100)**	**1 (100)**	**0**	**0**	**20 (100)**	**3 (100)**	**1 (100)**	**1 (100)**	**3 (100)**	**2 (100)**	**108**	**15**
**Total Unidentified Vertebrates**		**0**	**—**	**1**	**—**	**6**	**—**	**0**	**—**	**1**	**—**	**1**	**—**	**0**	**—**	**9**	**—**
Olivinae	olive snails	- -	- -	- -	- -	- -	- -	- -	- -	- -	- -	- -	- -	1 (12.50)	1 (12.50)	1	1
*Cassis* sp. (*madagascariensis*?)	queen helmet	- -	- -	1 (0.08)	1 (12.50)	- -	- -	- -	- -	- -	- -	- -	- -	- -	- -	1	1
*Prunum apicinum*	Atlantic marginella	- -	- -	16 (1.28)	16 (1.33)	- -	- -	- -	- -	1 (1.89)	1 (2.13)	- -	- -	- -	- -	17	17
**Total Marine Mollusks**		**0**	**0**	**17 (1.36)**	**17 (1.41)**	**0**	**0**	**0**	**0**	**1 (1.89)**	**1 (2.13)**	**0**	**0**	**1 (12.50)**	**1 (12.50)**	**19**	**19**
Unionidae	river clam	7 (11.67)	5 (8.62)	95 (7.62)	53 (4.40)	10 (20.83)	5 (11.63)	2 (14.29)	2 (14.29)	19 (35.85)	13 (27.66)	5 (55.56)	3 (42.86)	4 (50.00)	4 (50.00)	142	85
*Pachychilus* cf. *indiorum*	jute snail	- -	- -	- -	- -	- -	- -	- -	- -	1 (1.89)	1 (2.13)	- -	- -	- -	- -	1	1
*Pomacea flagellata*	Central American apple snail	48 (80.00)	48 (82.76)	1113 (9.07)	1113 (92.44)	35 (72.92)	35 (81.40)	11 (78.57)	11 (78.57)	20 (37.74)	20 (42.55)	2 (22.22)	**2 (28.57)**	2 (25.00)	2 (25.00)	1231	1231
**Total Freshwater Mollusks**		**55 (91.67)**	**53 (91.38)**	**1208 (96.95)**	**1166 (96.84)**	**45 (93.75)**	**40 (93.02)**	**13 (92.86)**	**13 (92.86)**	**40 (75.47)**	**34 (72.34)**	**7 (77.78)**	**5 (71.43)**	**6 (75.00)**	**6 (75.00)**	**1374**	**1317**
*Neocyclotus dysoni*	common crater snail	- -	- -	2 (0.16)	2 (0.17)	2 (4.17)	2 (4.65)	- -	- -	- -	- -	- -	- -	- -	- -	4	4
*Orthalicus princeps*	princess cone snail	- -	- -	1 (0.08)	1 (12.50)	- -	- -	- -	- -	2 (3.77)	2 (4.26)	2 (22.22)	2 (28.57)	- -	- -	5	5
*Bulimulus* sp.	terrestrial cone snails	2 (3.33)	2 (3.45)	14 (1.12)	14 (1.16)	- -	- -	- -	- -	4 (7.55)	4 (8.51)	- -	- -	- -	- -	20	20
*Euglandina* sp.	wolfsnail or marauder snail	1 (1.67)	1 (1.72)	1 (0.08)	1 (12.50)	1 (2.08)	1 (2.33)	- -	- -	4 (7.55)	4 (8.51)	- -	- -	1 (12.50)	1 (12.50)	8	8
cf. *Streptostyla* sp.	oval snails	2 (3.33)	2 (3.45)	- -	- -	- -	- -	1 (7.14)	1 (7.14)	- -	- -	- -	- -	- -	- -	3	3
Gastropoda, terrestrial	unidentified terrestrial snails	- -	- -	3 (0.24)	3 (0.25)	- -	- -	- -	- -	2 (3.77)	2 (4.26)	- -	- -	- -	- -	5	5
**Total Terrestrial Snails**		**5 (8.33)**	**5 (8.62)**	**21 (1.69)**	**21 (1.74)**	**3 (6.25)**	**3 (6.98)**	**1 (7.14)**	**1 (7.14)**	**12 (22.64)**	**12 (25.53)**	**2 (22.22)**	**2 (28.57)**	**1 (12.50)**	**1 (12.50)**	**45**	**45**
**Total Invertebrates**		**60 (100)**	**58 (100)**	**1246 (100)**	**1204 (100)**	**48 (100)**	**43 (100)**	**14 (100)**	**14 (100)**	**53 (100)**	**47 (100)**	**9 (100)**	**7 (100)**	**8 (100)**	**8 (100)**	**1438**	**1381**
**TOTAL**		**60 (3.88)**	**58 (4.15)**	**1309 (84.67)**	**1212 (86.82)**	**69 (4.46)**	**44 (3.15)**	**14 (0.91)**	**14 (1.00)**	**73 (4.72)**	**50 (3.58)**	**10 (0.65)**	**8 (0.57)**	**11 (0.71)**	**10 (0.72)**	**1546**	**1396**

In most cases, NISP represents the minimum number of elements in a context. Percentages are based on either total number of vertebrates or invertebrates per period. The final total percentages are based on the total NISP and MNI counts for all periods.

**Fig 5:**
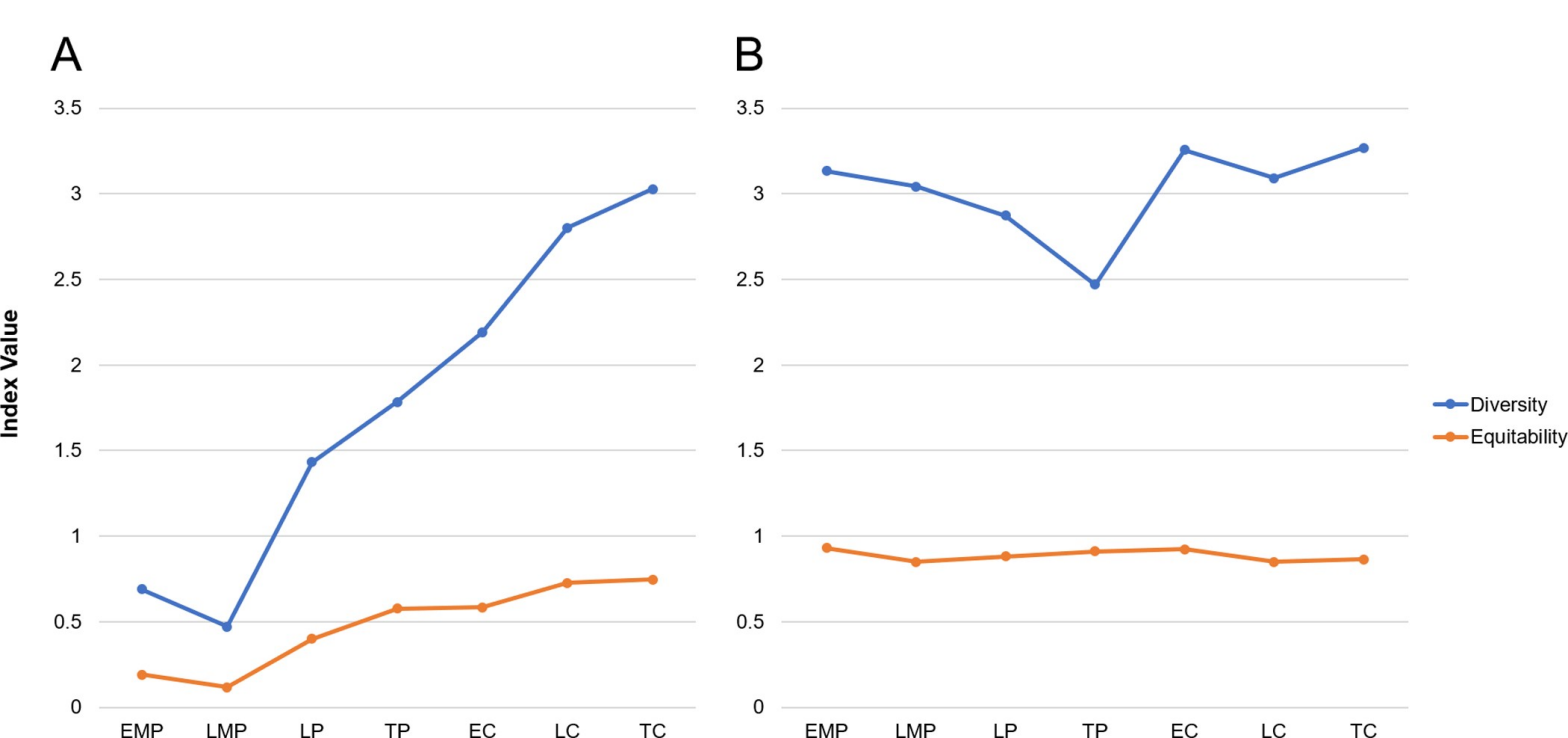
Taxonomic diversity and equitability at Ceibal and Caobal. (A) All animals at Ceibal and Caobal. (B) Diversity and equitability of only vertebrate specimens. Note MNI is used as the base of calculation. EMP = Early Middle Preclassic, LMP = Late Middle Preclassic, LP = Late Preclassic, TP = Terminal Preclassic, EC = Early Classic, LC = Late Classic, TC = Terminal Classic.

**Fig 6:**
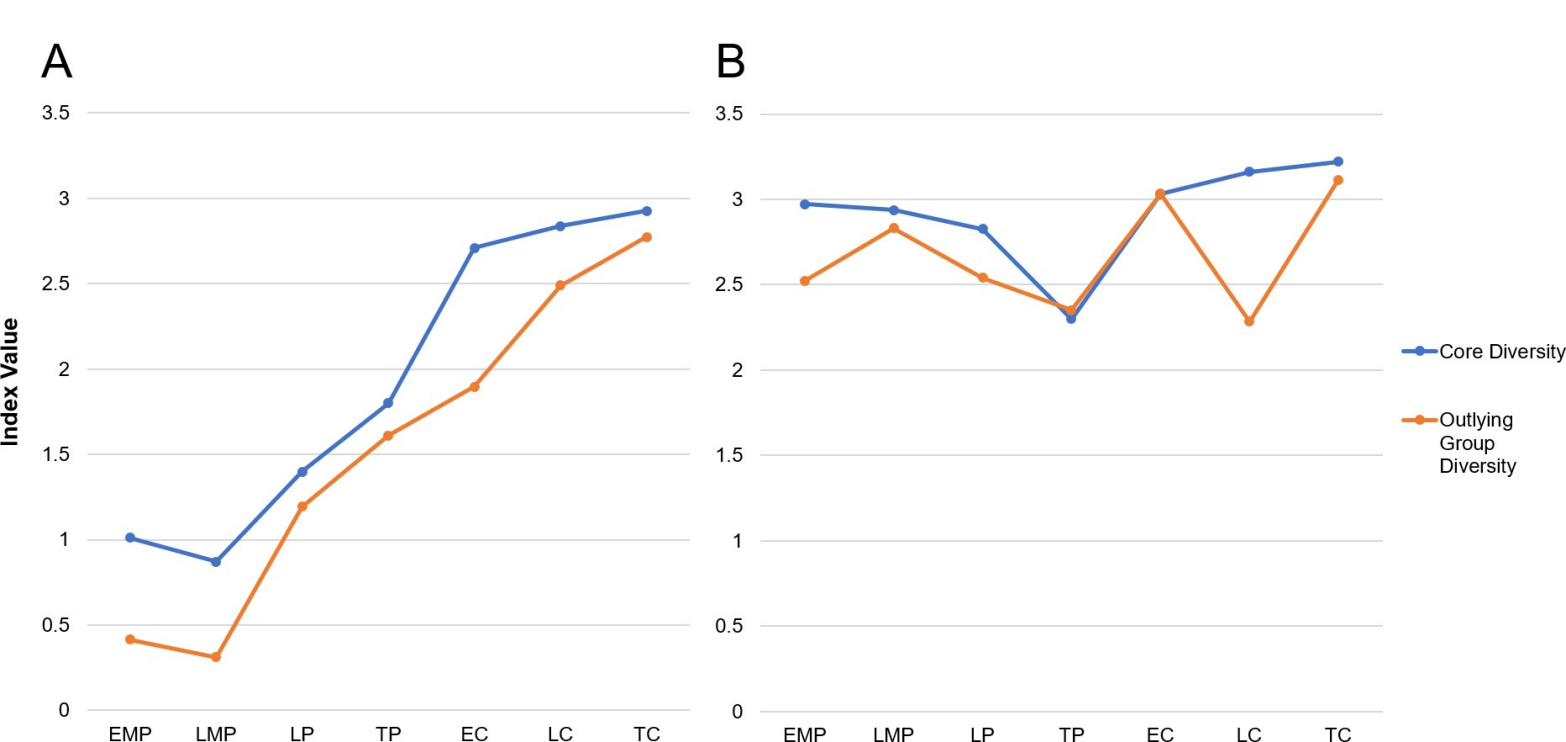
Taxonomic diversity in the ceremonial core of Ceibal (Groups A and D) and in the outlying residential and minor groups, including Caobal. (A) All animals at Ceibal and Caobal. (B) Diversity of only vertebrate specimens. Note MNI is used as the base of calculation. EMP = Early Middle Preclassic, LMP = Late Middle Preclassic, LP = Late Preclassic, TP = Terminal Preclassic, EC = Early Classic, LC = Late Classic, TC = Terminal Classic.

**Fig 7:**
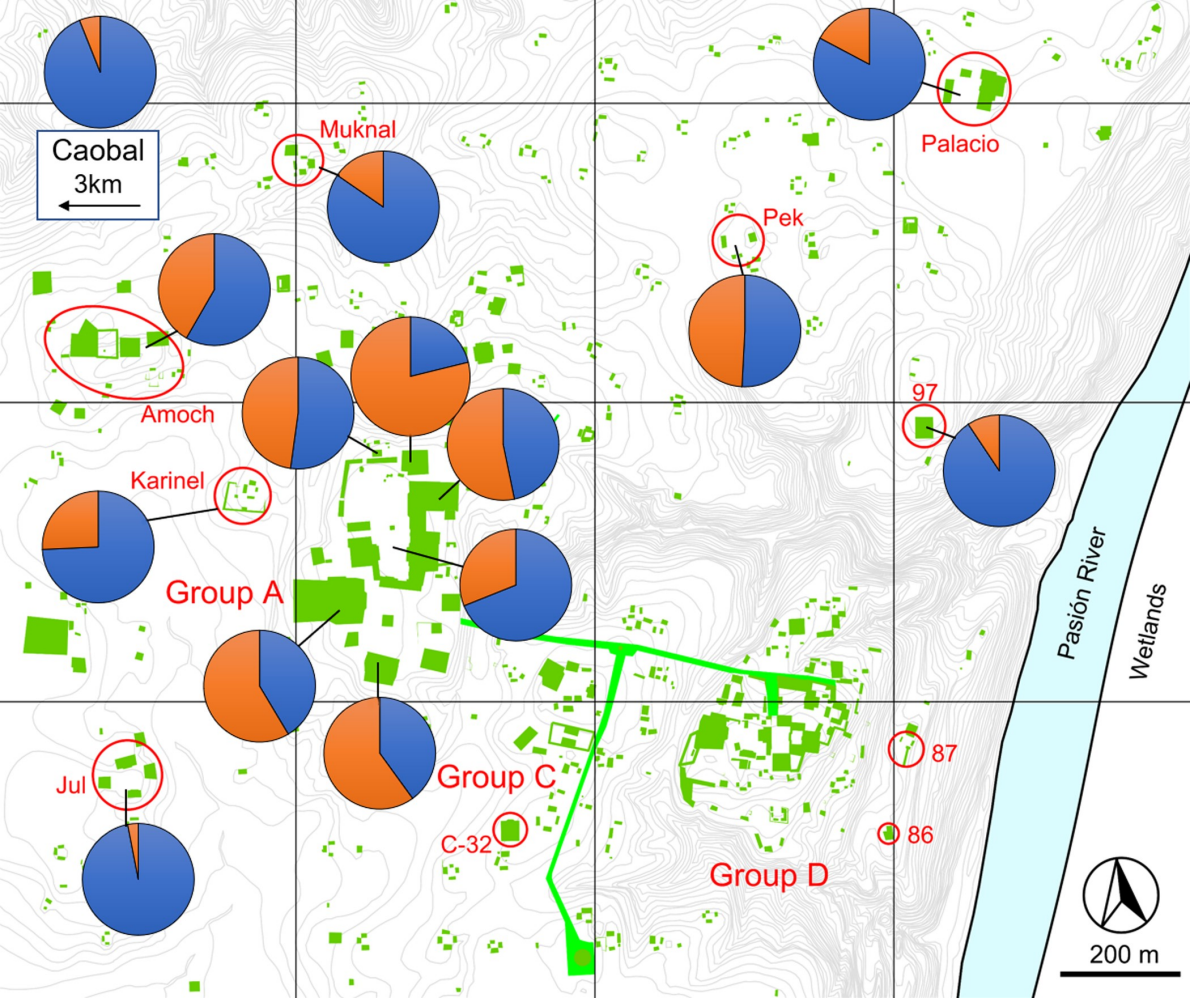
Preclassic fauna distribution across Ceibal (Middle and Late Preclassic periods). Blue is invertebrates, and orange is vertebrates. Map modified from [16].

**Fig 8:**
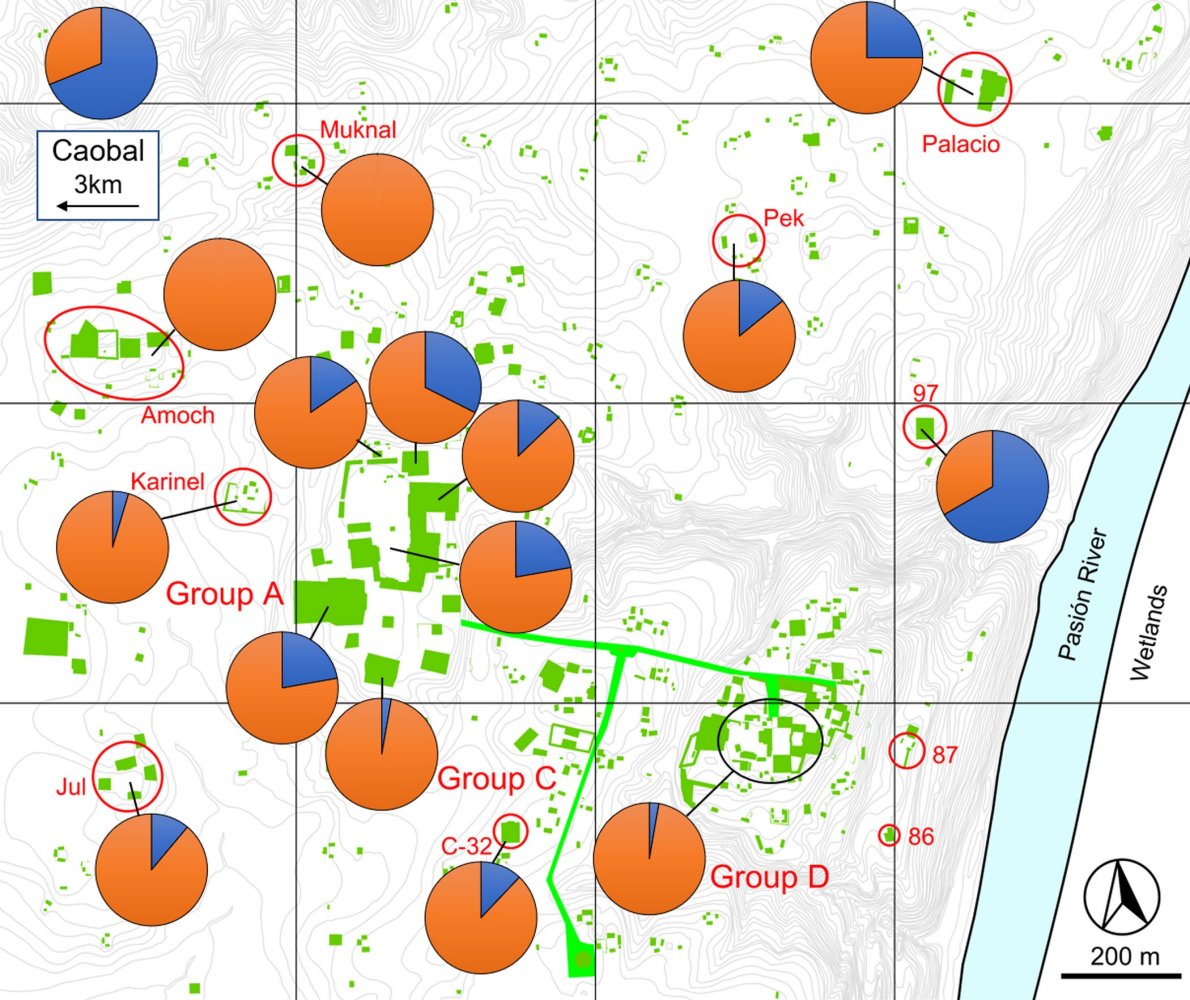
Classic fauna distribution across Ceibal (Early, Late, and Terminal Classic periods). Blue is invertebrates, and orange is vertebrates. Map modified from [16].

When assessing the diversity of both vertebrates and invertebrates across the site, invertebrates dominate the assemblage during the Preclassic phases, leading to a much lower diversity score (Figs 5 and 6). This shift between a predominance of invertebrates in the faunal record of the Preclassic period to vertebrates in the Classic period can be observed across the entire site (Figs 7 and 8). While it is partly attributable to dense concentrations of discarded freshwater mollusks found in some Preclassic deposits (discussed below), the Preclassic freshwater mollusks are prevalent in many different types of contexts, including middens, construction fill, human burials, and ritual offerings. Str. A-18 is the only exception to this trend, where there are more freshwater mollusks in the Classic phases than Preclassic. Invertebrates are always dominant at Caobal and Platform 97, but still decrease proportionally compared to the number of vertebrates between Preclassic and Classic periods.

Interestingly, when the invertebrate component of the assemblage is removed from the diversity calculation, the diversity score for the entire site’s vertebrate assemblage is fairly stable across time periods (Fig 5B). This does not mean that the same animal taxa are represented in all periods, as will be discussed in the next sections, but it does imply that the inhabitants of Ceibal hunted and fished a wide range of vertebrate taxa during all periods. We see this same trend when comparing the diversity for the ceremonial core area and the outlying residences and minor groups (Fig 6B). In both instances, the diversity factor is fairly high (for comparison with other archaeological sites, see [56]).

### Taxa-specific patterns: Invertebrates

There are always more freshwater invertebrates at Ceibal than marine mollusks, even during the Classic period when the number of freshwater mollusks decreases significantly. Unlike some of the Belizean archaeological sites where the gastropods known locally as *jute* (*Pachychilus* sp.) are found by the tens of thousands [57–61], these are infrequently encountered at Ceibal. The vast majority of freshwater taxa at Ceibal are apple snails and freshwater mussels. Both are found in much higher proportions (>50% of the total NISP and MNI) in the Preclassic phases than the Classic. Apple snails, in particular, dominate Ceibal’s faunal assemblage during the late Middle Preclassic period (700–350 B.C.), with a minimum estimate of 5752 snails found across the site, along with another 1113 at Caobal.

Marine taxa at Ceibal are diverse and variable through time. The majority of specimens appear to have been imported for ornamental uses, judging by the fact that over 90% of the marine mollusks were cut, carved, or pierced. Temporal patterns are strongly apparent, following regional trends where certain types of mollusks were imported and exchanged throughout the Maya lowlands for specific functions. Yet for the most part, Ceibal does not have many marine invertebrates (only 729 specimens) compared to other sites like El Zotz [62] or Tikal [63], likely due in part to its distance from the Caribbean and Pacific coasts (this was also noted by Willey [64] following the original Harvard excavations, where only 29 definitive marine shell pieces were recovered). The greatest variety of marine invertebrates appear during the late Middle Preclassic and Terminal Classic periods (13 and 10 species, respectively). The late Middle Preclassic and Terminal Classic periods are the two times when Ceibal had gained significant sociopolitical control in the area, particularly the Terminal Classic when Ceibal briefly became a regional capital. During these periods, it may have taken control of the local trade of these items.

### Taxa-specific patterns: Vertebrates

The vertebrate portion of Ceibal’s faunal assemblage exhibits many notable trends over time (Figs 3 and 4). There are proportionally more fish in the late Middle Preclassic (42.7% of vertebrates) and Early and Late Classic periods (34.5% and 72.0%), especially the latter, when they appear to dominate the assemblage. This is a pattern attributed to both overall proportion and actual number of skeletal elements recovered. The fish assemblage is still under analysis, but it appears that the entire assemblage is dominated by three groups: gar fish (*Atractosteus tropicus*), catfish (Siluriformes), and cichlids (Cichlidae); these three will likely still be the most common taxa after the analysis is completed. Notably, catfish and cichlids were the only fish identified from the Harvard excavations [29, 31]. Other fish, including snooks (*Centropomus* sp.) and freshwater drum (*Aplodinotus grunniens*), appear occasionally in the assemblage (15 and 21 bones, respectively). It is very likely that many of the smallest fish (vertebral diameter <3mm) that were recovered from fine-screening and flotation belong to the Poeciliidae family, of which there is a wide variety of species in the Usumacinta [40, 65, 66], but they will need to be compared with a thorough ichthyology collection for identification. Referring the MNI, there are about equal proportions of gar fish and cichlids in the Preclassic and Classic assemblages (NISP tends to overquantify gar fish in assemblages due to their numerous boney scales), but the catfish species fluctuate in abundance over time. While smaller varieties of catfish, namely in the *Cathorops* genus, are found in most time periods, only during the Preclassic period does a larger species of catfish appear. Based on several large otoliths found in Middle Preclassic phases, the fish is comparable in terms of size and shape with large sea catfish (*Ariopsis felis*), but may in fact be the large Usumacinta catfish (*Potamarius usumacintae*; [67]).

Regarding the reptiles and amphibians, there are very few (NISP = 105, MNI = 20) of the latter across the site in all time periods, but reptiles are consistently common in most deposits. The majority of reptiles are turtles (95.8% NISP, 75.4% MNI), which include at least five identified species: the Central American river turtle (*Dermatemys mawii*), the slider (*Trachemys venusta*), the Central American snapping turtle (*Chelydra rossignonii*), the Mexican giant musk turtle (*Staurotypus triporcatus*), and the small mud and musk turtles (*Kinosternon* sp.). With the exception of the snapping turtle, these are the same species identified by Pohl [29, 31] from the original Harvard excavations. The proportions of these taxa are fairly consistent across the time periods, with the notable exception of the *Dermatemys* river turtle, a large-bodied individual that becomes much more common across Ceibal during the Classic period (Preclassic NISP = 108, Classic NISP = 883). Pohl [31] had also noted the large quantity of *Dermatemys* bones from the Harvard excavations, where they had made up 22% of the total identified fauna. Other reptiles at the site include crocodiles or caimans (Crocodylidae), iguanas (Iguanidae), snakes, and small lizards (MNI = 10) whose remains frequently appeared in the fine-screen collections. The latter still need careful identification with a comprehensive herpetological collection.

Bird remains generally do not preserve well in the Guatemalan tropics, although several bones (NISP = 295, MNI = 77) were found at Ceibal, spanning all time periods and a number of different locations. The majority of bird remains were fragmentary, and usually found singly, rather than as a part of a skeleton, thus contributing to the low NISP but high MNI values compared to other animal classes. Species vary widely across time. Aquatic birds are found in most periods, ranging from ducks to coots to herons (e.g., *Dendrocygna* sp., *Egretta* sp., *Nycticorax nycticorax*), which is to be expected considering Ceibal’s location by a river. Only one definitive passerine bone was recovered (a tarsometatarsus of a great-tailed grackle, *Quiscalus mexicanus*), despite their prevalence at the site today, and were not even recovered through flotation. The most significant trend concerns the turkeys, which appear at Ceibal in the Early Classic period and steadily rise in number until the Terminal Classic. Ancient DNA analysis was performed on three of these Late and Terminal Classic birds (courtesy of Camilla Speller, University of British Columbia), and resulted in the identification of both ocellated and wild/domesticated species (*Meleagris ocellata* and *gallopavo*, respectively) at the site. Pohl [29, 31] had also identified seven ocellated turkey bones in exclusively Late Classic deposits.

There is a wide variety of mammals in the assemblage (at least 27 species), exhibiting more diversity than the other taxonomic classes; however, this is likely due to the fact that mammal taxa in Guatemala are more commonly represented in comparative collections and their bones tend to preserve more than other animal classes. Although the mammals range from felines to anteaters to peccaries, the majority are dogs and deer (NISP = 18.4% and 13.0% of vertebrates, respectively). Domestic dogs (*Canis lupus familiaris*) make up the majority of identified mammals during the Preclassic phases (NISP = 467, MNI = 50), which lack mammal diversity compared to the Late and Terminal Classic period. Several partial dog skeletons were recovered from the Early Classic period residential platform of Karinel, contributing to the large proportions of dogs during that period. But during the Late and Terminal Classic, deer become the more dominant taxa (of mammals identified below level of class, NISP = 58.7% or 551 bones, MNI = 31.8% or 67 individuals), and the number of dogs significantly declines (NISP = 95; MNI = 19). The greatest diversity of mammals also occurs during these later periods. These general trends were also found in the original study by Pohl [31] when analyzing the Harvard fauna, where 19 mammal species were found in the Late Classic period, all of which overlap with those identified in Ceibal’s Late and Terminal Classic deposits of this study. Deer made up 50.2% of the Late Classic bones in that original faunal assemblage (NISP = 295; MNI = 28).

## Discussion

The following section focuses on the most noticeable trends across Ceibal, and compares these with reported data from other regions of the Maya lowlands and elsewhere. The two most significant aspects of the Ceibal dataset are its large number of specimens and long occupational record, which allows for an examination of trends noted or suspected at other sites, where datasets either lacked a large number of specimens or both Preclassic and Classic components to compare.

### The classic period decline of freshwater mollusks

**Fig 9:**
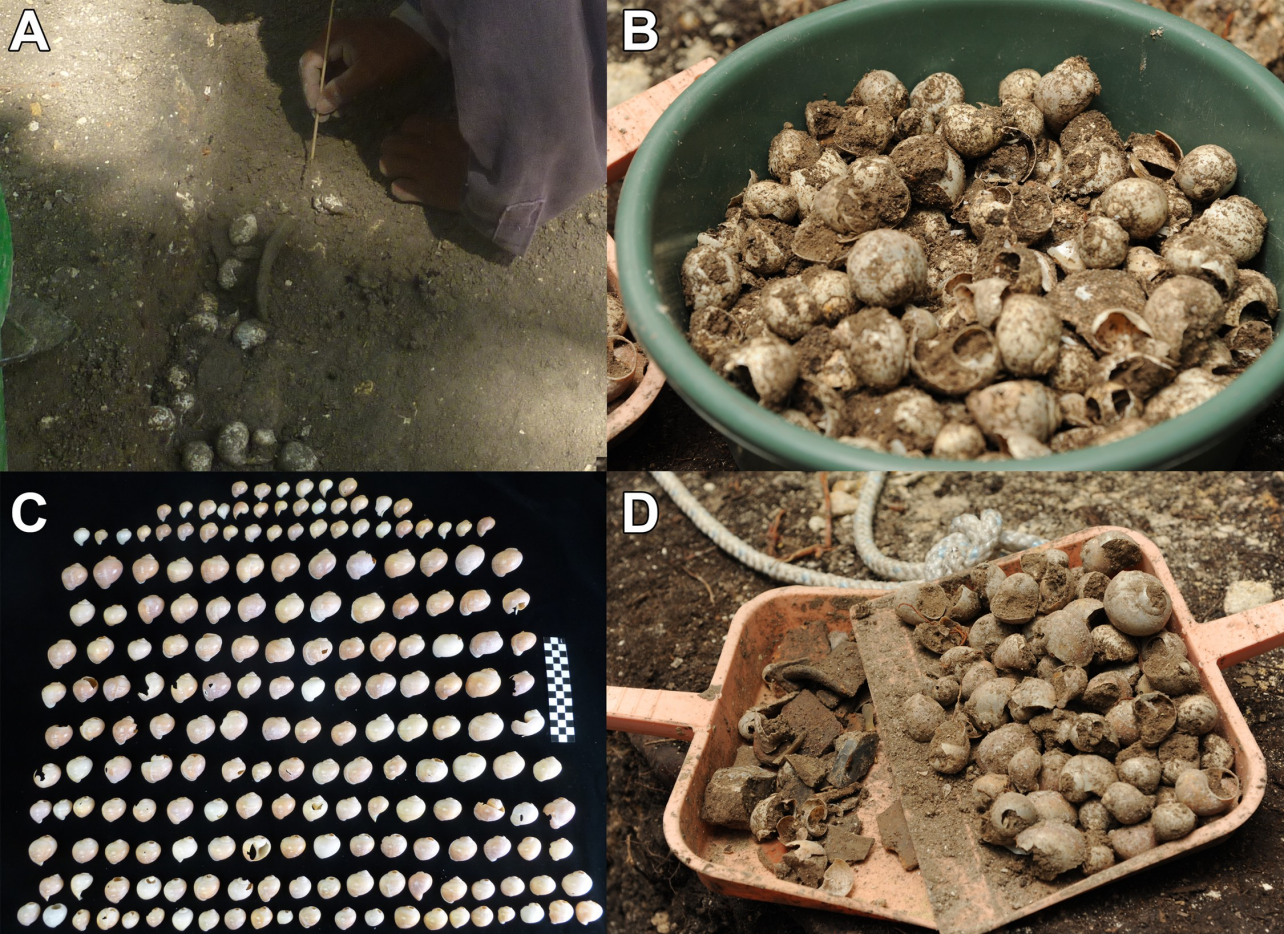
Examples of Middle Preclassic apple snail (*Pomacea flagellata*) deposits. (A) Karinel Group, operation CB211C-13-6-3. (B) Jul Group, Burial 126, operation CB210A-2-6-4 and 2-6-5. (C) Caobal, operation AN1A-1-19-1. (D) Jul Group, Burial 126, operation CB210A-2-6-4 and 2-6-5. Photos (A) and (C) by Sharpe, photos (B) and (D) by Burham.

The dense deposits of freshwater mollusks across Ceibal and Caobal during the Preclassic period include both apple snails and freshwater mussels, although the former are found in considerably greater abundance (Fig 9). The greatest concentrations of these invertebrates date to the late Middle Preclassic period (700–350 BC), although dense deposits are still occasionally found in earlier and later Preclassic phases. There is only one species of apple snail present, although the ranges in size are often significant, from ~1–8 cm in diameter, even within a single deposit. There is no sign of cooking or discoloration from heating on the surface, nor uniform piercing to extract the gastropod. This coincides with what previous studies have reported in the Peten [68]. It would appear that they were collected in dense quantities, regardless of size, and that the gastropods inside were removed in such a way that the shells were undamaged. Light boiling may have helped remove the snails from their shells without damaging the shell itself, but this would need to be verified through an experimental study. Apple snails are still occasionally eaten today in northern Guatemala and Belize, and local residents who live in and around the modern town of Sayaxche claim they can be found in greater abundance during the end of the rainy season and start of the dry season (December-January) when they emerge from the mud to mate (this has also been noted in Belize; Emery pers. comm.). It may be that the dense deposits of apple snails are evidence of a seasonal activity. However, the life cycle of the Mesoamerican apple snail has not been adequately investigated, so as of right now this intriguing possibility is uncertain.

Whereas apple snails prefer slow-moving water and can be found in both the marshy areas of the Pasión River as well as shallow lakes and *aguadas* (small ponds), the freshwater mussels can only be obtained from the river. Identification of the mussels to species level is currently ongoing, since a phylogenetic study has only recently been performed to assess the diversity within the Usumacinta family of Unionids [42, 43]. However, the majority of mussels at Ceibal appear to belong to the species *Psoronaias semigranosa*, which is most commonly found today along the riverbank near the site. It is still frequently eaten by the people living around Sayaxche, including many of the Guatemalan excavators working on the Ceibal project, and is usually not cooked before consumption.

Unlike other Maya sites [57–61], especially those of Belize, there are very few *jute* snails at Ceibal. With the exception of a concentrated deposit of 75 *jute* snails in the Karinel residential group dating to the early Middle Preclassic period (1000–700 BC), they are only found occasionally at the site. It may be that they were not common in the river near Ceibal. Examination of the riverbank and a nearby lake during the 2014–2015 excavation seasons did not locate *jute*, although both apple snails and river mussels were encountered.

The massive shift in freshwater snails and mussels to predominantly vertebrates between the Preclassic and Classic periods raises a number of important questions regarding the history of Ceibal, its surrounding environment, and the culture of subsistence strategies in general throughout the lowland Maya region. Significantly, this temporal invertebrate-vertebrate shift has been reported previously at other Maya sites, especially those with large faunal assemblages covering both the Preclassic and Classic periods. Moholy-Nagy was the first to report this trend at the large site of Tikal in the eastern Peten [68, 69], noting that dense deposits of apple snails were only found during the Preclassic period. Similar patterns have been identified at Barton Ramie [70], Blue Creek [71], Cahal Pech [72], Cuello [73, 74], K’axob [75], Pacbitun [76], San Bartolo-Xultun [77, 78], and Yaxha-Sacnab [79], among others. At nearly all of the Belizean sites, the river mussel *Nephronaias* sp. was also found in abundance (see [80] for a regional overview). At Ceibal, K’axob [75], and Nohmul [81], deposits of apple snails were found with human burials. Burial 126 (c. 700–450 BC) at Ceibal, found in the residential Jul Group, contained the extended burial of an adult male placed at the bottom of a carved-out depression in the limestone bedrock that had been filled with hundreds of apple snails. Lucero and Kinkella [81] believe such deposits may be a symbolic reference to the Maya belief in an aquatic afterlife. However, dense deposits of snails are often found without humans remains at Ceibal and other sites; it may be they are the remains of a minor feasting event, and perhaps in some cases, as in that of Burial 126, a feast in association with the burial itself. Burham [82] noted that some of the snails appeared to be placed with the burial, including a few under an upturned plate on the individual’s knees, while others where intermixed with the fill and were interpreted as midden refuse. Similar lowland burials from the late Middle Preclassic period have been found under midden refuse at other sites, including Chiapa de Corzo and Tikal [83], which is the likely explanation for the majority of the apple snails in this deposit.

What happened to the apple snails and river mussels between the Preclassic and Classic periods? Did the change in subsistence have a sociocultural explanation, or an environmental one? A number of hypotheses have been proposed over the years to explain this pattern. Two of the first to notice this trend, Moholy-Nagy [68] and Willey [70], suggested that rising human populations resulted in the overexploitation of these freshwater mollusks (but not the *jute*). Others [84, 85] suspected massive land-use changes over time in Belize may have caused these trends, mainly due to changing agricultural practices between the Preclassic and Classic periods, resulting in the draining and desiccation of wetlands that created an uninhabitable environment for the apple snails. Furthermore, a number of paleoecological studies have found evidence for an increased period of drought toward the end of the Late and Terminal Preclassic periods [8, 86, 87], which may have diminished wetland habitats and encouraged the lowland Maya inhabitants to shift their procurement strategies to other species besides apple snails.

Based on modern ethnographic observations of how Lacandon Maya women were using the *jute* shells in the 1960s and 70s, Nations [88] postulated that perhaps the trend observed in the apple snails was due to the ancient Maya pulverizing the shells for the use of lime production and to supplement their diet. Baer and Merrifield [89] also describe this practice, although the snail species is not specified. Since *jute* snails are still present in abundance in the Belizean Classic period sites, and the disappearance is most noticeable in apple snails and river mussels, evidence to support this third theory is lacking.

These theories were developed several decades ago, based primarily on data from Belize and Tikal, where the majority of excavations of the time were conducted. The new datasets from Ceibal and Caobal are important in this regard, because they represent a region of the Maya lowlands that lies far to the southwest of the previous studies, and in an entirely different watershed (the Pasión and Usumacinta). Although there is much evidence for large-scale landscape transformations at Ceibal, such as the Middle Preclassic artificial platform where Group A is located [26], these changes do not temporally coincide with the decline in mollusks at the site. The fact that river mussels, which differ considerably in both species and size on the Belizean/eastern Guatemalan and Usumacinta sides of the Maya lowlands, also conform to this trend implies that the change affected mollusks harvested from entirely different habitats. While there are Classic period river mussels at Ceibal, many are modified in the form of ornaments, and their numbers are only about 20% of the Preclassic mussel numbers. At the nearby Late Classic period site of Aguateca, for example, hundreds of river mussels were recovered on the floors of structures where they were being worked into ornaments or used as inkpots (Aguateca was quickly abandoned before an attack by an unknown enemy, and so the mussels were left in-situ where they had been worked on [56, 90]). Thus, this is an indication that the mussels were still in the river, but why were they no longer a major component of the Classic period subsistence base?

There is no clear explanation at the moment, but there is no doubt that the change in subsistence strategies was strongly influenced by significant sociocultural shifts that took place between the Preclassic and Classic periods. Clearly the change was widespread, affecting Ceibal as well as settlements all the way to the Belizean coast. It is also observed in a number of different context types at Ceibal, Caobal, and at other sites, ranging from construction fill in residences and ceremonial areas to special deposits like burials. Thus, it is not a product of depositional bias. It may be the result of a change in dietary preference, in which the mollusks were not given the edible preference they once had, but it seems unusual that the Classic period Maya would intentionally forgo such a readily-accessible resource. However, it may be that the elites who inhabited the monumental site cores and closest residential groups did not consume freshwater invertebrates. Lower-class and more “rural” citizens, whose residences were likely not located near the ceremonial center and were not a target of excavation by the CPAP, may have been consuming mollusks more frequently; we simply do not have a comparable sample of their faunal assemblage to assess. There is some evidence that freshwater mollusks were more commonly consumed by the lower-class inhabitants of Classic period cities [56, 91]; however, it is an area where much more work needs to be done.

### Marine imports to Ceibal

Marine imports to Ceibal included at least 19 species of marine mollusks (Table 2; [28]), as well as two sea urchins (cf. *Eucidaris* sp.), and one large bull shark tooth (*Carcharhinus leucas*). The two peak periods of marine species diversity were the late Middle Preclassic and the Terminal Classic periods. The first of these was a period of great expansion in terms of population and construction activities at Ceibal, when it became one of the dominant Preclassic ceremonial centers in the lowlands. The Terminal Classic period was much shorter, but significant in that Ceibal became one of the last regional capitals in the Peten. Almost all of the marine specimens that could be identified to species were from the Caribbean Sea or the Gulf of Mexico (although as noted in [28], there is a photo of a Classic period Pacific *Oliva porphyria* in the Harvard excavation reports [64]). During this time, Ceibal had strong ties with the lower Usumacinta region and the Gulf coast, but it is not clear if the marine mollusks came from that direction or from Belize. Specimens that could not be identified to species level, and even some globe-spanning species like the bull shark, could possibly come from either the Pacific or Atlantic sides. However, since the identifiable species are primarily Atlantic taxa, and can also be found at many central Belizean sites, it seems likely that the major overland trade routes for marine mollusks to Ceibal came through Belize during both Preclassic and Classic periods, with possibly some coming from the Gulf area. Almost all of the marine shells exhibited signs of cut marks, piercing, and carving, indicating they were primarily imported for use as ornaments.

Besides the bull shark tooth, no marine fish have been clearly identified at Ceibal. Some of the snook (*Centropomus* sp.) bones and large catfish (Ariidae) remains may have been marine imports from the coast, but both fish are found in the Pasión river today [40, 67]. There are also no stingray spines, which are ubiquitous at other Classic period sites in the lowlands, including nearby sites like Altar de Sacrificios [92] and Aguateca [90]. Other marine fish bones, particularly parrotfish (*Sparisoma* sp.), grouper (Serrenidae), and hardhead catfish (*Ariopsis felis*), have been found at inland Maya centers, including Cahal Pech [72], Caracol [93, 94], Holmul [77], Lubaantun [95], Mayapan [4, 96], and Tikal [97]. It is possible that the lack of Pacific species, ray spines, and other important Classic period marine imports found at many other major centers was due to Ceibal’s role as a vassal to Dos Pilas for many years [12]. Only during the end of the Terminal Classic did Ceibal briefly become a capital center, at a time when most inland trade routes were in disarray as sites throughout the southern lowlands were abandoned.

### The freshwater fish

During the Middle Preclassic and Early/Late Classic periods, fish were the dominant vertebrate taxa at Ceibal in terms of NISP. Future analyses of the fish assemblage with a comparative freshwater fish collection from the region will likely reveal that they are also the dominant class in terms of MNI. Fish are found across the site, which is unsurprising considering Ceibal’s location directly on the edge of the Pasión River. The numerous tiny fish bones, which may belong to the small-bodied and extensive Poeciliidae family [40, 65], were likely caught with nets. Larger fish, including catfish, snooks, and especially the armor-plated gars, would have required spears. There is no clear evidence of anything resembling a fishhook at Ceibal, although bone hooks have been found elsewhere in the Pasión region [92, 98].

Most of the fish were likely caught locally, and can be found in the river today. The tropical garfish (*Atractosteus tropicus*), in particular, prefer slow-moving rivers like those where the Pasión passes near Ceibal [99]. There are many catfish, including members of the genus *Cathorops*, that live in the Pasión today. Although snook (*Centropomus* sp.) are often associated with marine or estuarine waters, they can swim far upriver and are found in the Pasión near Ceibal [40]. The freshwater drum (*Aplodinotus grunniens*) has the widest geographic distribution of any freshwater fish in North America and can be found in both slow and fast-flowing rivers [100, 101]; thus, the drums in the assemblage were also likely caught near Ceibal.

The taxonomy of the fish species in the Usumacinta River and its tributaries is still not well defined; this is a longstanding problem in Maya zooarchaeology, forcing many analysts to classify fish elements to broad family and order level categories. This does little in the way of providing information regarding the habitat where specific fish were hunted, how fish behavior influenced how they were caught, or the emic classification and recognition of fish species among the ancient Maya. For example, there are some 30 species of cichlids reported in Guatemala today [40, 102, 103], and their taxonomic identifications have changed frequently in recent years [104]. Their behaviors, shapes, and colors vary widely, which was likely noted by the ancient Ceibal fisherfolk. Two of the most common cichlids found in the Usumacinta River today are the *Petenia splendida* and *Mayaheros urophthalmus*, which are very likely represented in the Ceibal assemblage. In fact, of the two fish found in the original Harvard material, one was tentatively identified as a *Petenia splendida* (the other being a catfish) [31].

There are no obvious indications of changes among fish taxa over time at Ceibal; however, there are some patterns observed in terms of quantity of fish bones and location. Fish are found at all parts of the site in roughly even distributions during the Preclassic phases, but during the Late Classic period there are dense concentrations of fish bones in animal bone middens found in three separate locations: Str. A-2 near the ceremonial core, Group D’s East Plaza, and the Karinel Group. Identified fish in these deposits are very similar, and include cichlids, catfish (mainly *Cathorops*), gar, and freshwater drum. These dense concentrations of hundreds to thousands of fish bones indicate that, while freshwater mollusks no longer contributed to any significant degree in the elite diet, fishing was a mainstay of the Classic period subsistence base.

### The turtles

The majority of reptiles at Ceibal were turtles, which is generally very common at many lowland Maya sites [31, 56, 105–109]. Turtles were likely easy to capture, especially the sliders that tend to congregate in ponds and at swamp edges. There is clearly a preference for certain turtles at Ceibal, with the slider and Central American river turtle leading, and the snapping turtle being generally avoided, perhaps intentionally due to its demeanor; in fact, snapping turtles are absent or uncommon at most Maya sites. Only six bones, belonging to two individuals, were found at Ceibal. Several midden deposits at Ceibal included examples of mixed turtle species, often three or more.

There is a notable increase in Central American river turtle remains across Ceibal when comparing the Preclassic and Classic phases. The largest freshwater turtle in Mesoamerica, the *Dermatemys* is critically endangered today [110–112], and most attempts at conservation have been unsuccessful, since they are considered a delicacy and are very easy to hunt during the dry season when water levels are low. It seems unusual, then, that they are uncommon in the Preclassic, particularly during the late Middle and Late Preclassic periods when Ceibal reached one of its peak populations. One possible explanation for this may be that the turtle was actively transported throughout the Peten area during the Classic period, as has been suggested by biologists who found disjunct populations of related *Dermatemys* sharing a unique haplotype across the Maya region [113, 114]. There is a notable genetic affinity between *Dermatemys* populations in the Isthmus of Tehuantepec area and the Salinas River near the Pasión with those of the Sartsún River of the southern Belize/Guatemala border region that does not seem possible without humans having actively transported the turtle at some point. The turtle was clearly already in the region and hunted during the Middle Preclassic based on evidence from Ceibal, but the very low numbers suggest they were not common. The presence of limb elements among the Preclassic remains, although few (NISP = 5; see S1 Table), suggests that the Preclassic *Dermatemys* were whole animals and not imported carapaces. It would seem that the Classic period inhabitants had begun to either focus their procurement strategies on this one taxon, or they were managing and moving the turtles along the river. Classic period sites elsewhere in the Usumacinta area also have reported large quantities of these turtles [56], but there are few faunal reports from Preclassic period sites in the area with which to compare. Future ancient DNA research on the Ceibal turtle remains may reveal evidence for this hypothetical turtle transportation event by comparing the genomes of Preclassic and Classic individuals, and whether the Isthmus of Tehuantepec haplotype became more common over time.

### The birds: Preservation and domestication

**Fig 10:**
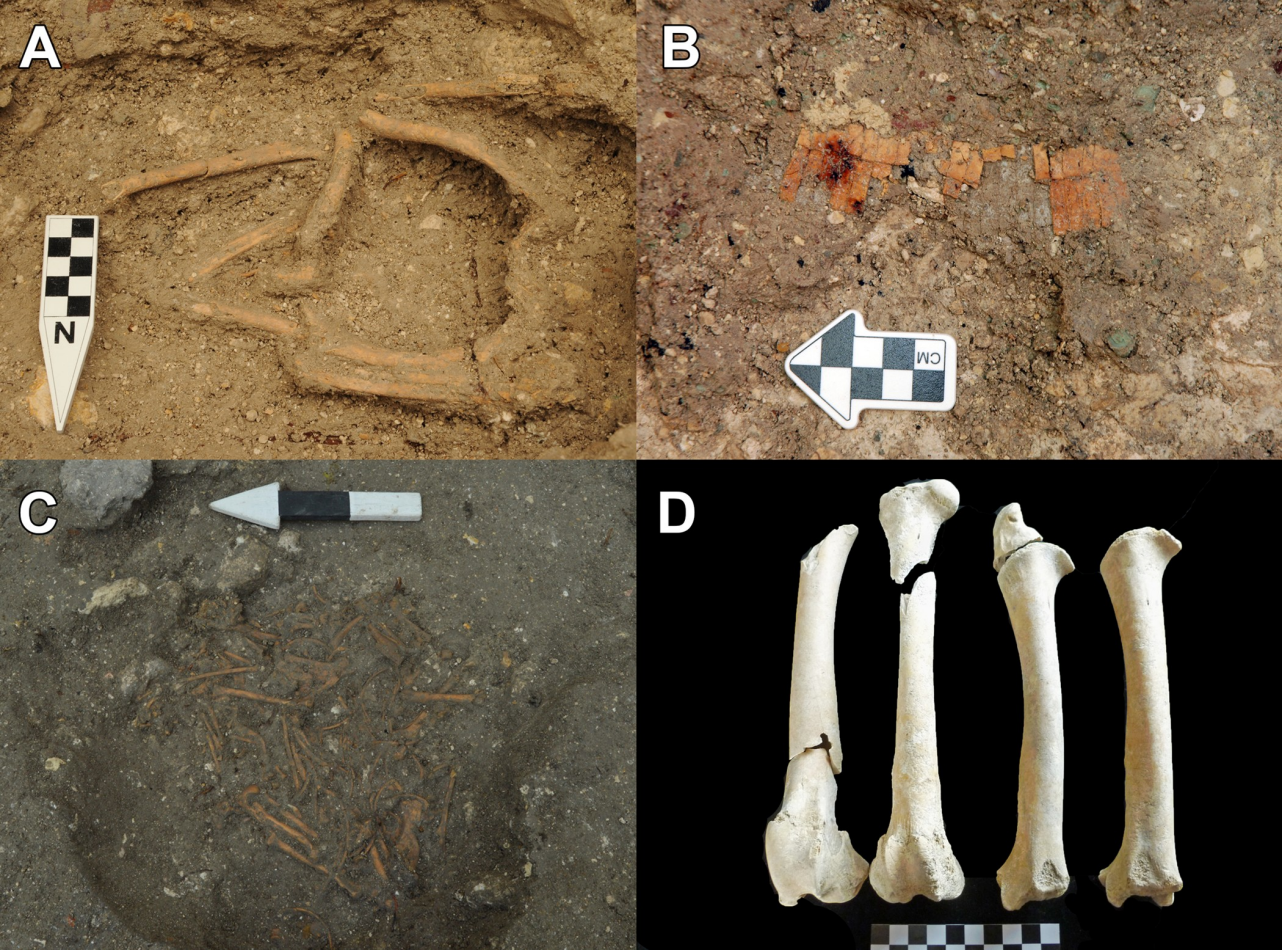
Examples of animal deposits at Ceibal. (A) Possible vulture (Cathartidae), late Middle Preclassic Group A Central Plaza, operation CB203B-18-6-7. (B) Early Middle Preclassic armadillo shell (*Dasypus novemcinctus*), Group A East Court, operation CB201F-3-12-5. (C) Early Classic burial of two dogs (*Canis lupus familiaris*), Karinel Group, operation CB211C-12-7-4. (D) Multiple deer (*Odocoileus virginianus*) femora from a Late Classic Group D midden in the East Plaza, operation CB208A-1-4-9 and 1-4-10. Photo (A) by Pinzón, photo (B) by Triadan, photo (C) by MacLellan, and photo (D) by Sharpe.

The number of bird remains at Ceibal is never very high, due in part to their poorer ability to preserve than other vertebrate classes, but a number of taxa could still be identified. The majority of birds are aquatic, including ducks and herons, and were likely hunted within close proximity to the site. There is an unfortunate dearth of small bird bones, which is particularly surprising considering the large number of tiny fish bones that were recovered through flotation. It would seem that, like small reptile and amphibian bones, bird bones did not preserve well. The recovery of a partial raptor bird skeleton (tentatively identified as a vulture, Cathartidae; Fig 10A) in the Middle Preclassic phase of the Group A Central Plaza suggests that under the right conditions, bird bones can be preserved at Ceibal for thousands of years. Unfortunately, this was not the case for the majority of bird specimens.

During the Classic period, there is a sudden increase in the number of turkey bones across the community (this was also noted in Pohl’s original analysis [29, 31]). Ancient DNA analysis on three turkey bones from excavations in front of the Late/Terminal Classic Group A palace (the East Court), the East Plaza of Group D, and the Karinel Group revealed a mix of both ocellated and “wild” (also known as “northern”) turkey species (*Meleagris ocellata* and *gallopavo*, respectively). The presence of the *gallopavo* species during the Classic period supports the hypothesis that they may have been introduced from central Mexico during the Terminal Preclassic period [115], and that true domestication of this species occurred sometime during the Late/Terminal Classic period as it was spread by humans throughout Mesoamerica [116]. It is unclear if both species were raised together in captivity at Ceibal, since an isotopic study of the turkey remains, including the confirmed *gallopavo* and *ocellata* specimens, revealed that the two *gallopavos* had maize-based diets while the *ocellata* specimen did not [117]. If the Late/Terminal Classic ocellated turkeys were wild, it seems unusual that there are virtually no ocellated turkey bones in the Preclassic period (with the exception of one unidentified turkey from the Terminal Preclassic period at the Karinel Group). Perhaps active rearing of the *gallopavo* turkey, a practice initiated originally in central Mexico and brought to the southern Maya area, may have encouraged rearing or at least loose husbandry of the *ocellata* species. Götz and colleagues [118] have noted from ethnographic interviews of local informants in the Yucatan that ocellated turkeys do not survive long in captivity near human enclosures, unlike the *gallopavo* species, so it is likely that they were ever intensively managed.

### The mammals

Mammals were the class with the greatest variety of species recovered at Ceibal. This is likely due to the better preservation of their bones compared to thinner-boned animals like birds, smaller reptiles, and amphibians. The majority of mammals at Ceibal were the white-tailed deer (*Odocoileus virginianus*) and domestic dog (*Canis lupus familiaris*). These two species were common across most phases of Ceibal’s history; in fact, it is very likely that the majority of unidentified mammal bones, which were mostly splintered or eroded fragments lacking diagnostic characteristics, were dogs and deer. Dogs represent the principal domestic animal in the Maya area, possibly having been introduced to Central America along with nomadic groups traveling south after the last Ice Age. Deer are one of the few large-bodied mammals on the Mesoamerican landscape, and are still fairly ubiquitous; it is unsurprising they were a prime target for Maya hunters. However, the role of dogs, deer, and other mammals appears to have varied over time.

#### The domestic dog: Friend or food?

There is little direct evidence that dogs were used as food at Ceibal, although it is likely that at least some dogs were consumed based on historic records from early Spanish colonists (e.g., [119]). Of the 1214 dog bones at Ceibal, 14 were burned and only 4 had cut marks that appeared to be signs of butchery rather than artifact manufacture. Most of the burning and all of the butchery marks were from remains found during the late Middle Preclassic period, indicating that some dogs during this period may have been eaten. A recent study [120] comparing the dog bone data from over a hundred Maya archaeological sites has found similar evidence corroborating the idea that dogs were used for a variety of roles, and that cut marks and burning are generally infrequently identified on dog bones, the main exception being drilled teeth for ornaments [93, 121].

Dogs were found in almost every operation at Ceibal and at Caobal, both in the fill of ceremonial structures as well as outlying residential areas. They were most often found as partial or scattered remains, rather than entire skeletons. Most dog bones in the Middle and Late Preclassic periods came from fauna-dense midden deposits, again suggesting that at least some dogs during this period were consumed. A deposit of two nearly-complete dog skeletons, a full-grown adult and a puppy with nearly all unfused bones, were recovered from the Karinel Group (Fig 10C); although they were originally believed to have dated to the early Middle Preclassic period, a radiocarbon date on the bones showed that they were an intrusive burial from the Early Classic period (398–539 cal. AD), a time when there were comparably few other dog bones found at Ceibal. In general, dog “burials” are very uncommon at Maya sites, and since these two were of different ages, it is possible their burial was a unique household offering.

Dogs’ sizes varied considerably, suggesting that there were a number of different morphotypes, or perhaps even breeds, present. This mix of distinctly large and small adult dogs has been noted at other sites previously, such as Colha and Cerros [122, 123]. A combined morphometric, isotopic, and genetic study of the Ceibal dogs is underway to determine the relationship between possible morphotypes and diets at the site over time. An isotopic study at Ceibal [117] showed that dog diets varied slightly over time, although they all consumed maize. It also identified two Preclassic dogs, found in the ceremonial site core, whose strontium and oxygen isotopes did not match the southern lowlands and which may have come from the Guatemalan highlands. These dogs were adults, and again suggest that not all dogs were consumed, but may have had a variety of roles over time. Dogs found at other archaeological sites, including Colha, Copan, Cuello, Lagartaro, and Tikal, have also been isotopically tested and found to have consumed varying quantities of maize during both Preclassic and Classic periods, indicating that perhaps some were intentionally fed maize, but others may have scavenged for food discarded in and around human settlements [124, 125].

#### Deer: A Classic period icon

While deer were always common at Ceibal, there are significantly more deer remains in the Late and Terminal Classic deposits than during earlier times (NISP = 228 and 287, respectively, in comparison to less than 100 during all earlier phases). This trend is observed in both white-tailed deer and brocket deer (*Mazama* sp.) species, although it is much more obvious in the former. White-tailed deer frequent a wider variety of habitats than brocket deer, and are attracted to agricultural fields, making them an easier target. Brocket deer tend to avoid cleared areas and stay in dense forests [126, 127].

The increase in deer bones from the Preclassic to Classic period has been observed before by zooarchaeologists working at other sites [91, 105, 128–132], and also in the earlier Ceibal excavations [29, 31]. The deer appears to have become a favored food during the latter part of the Classic period. There is evidence to believe that it held a special role during feasting events [130], and its bones, antlers, and hide were frequently used for crafting costumes and tools. At some of the largest Classic period centers where distinct social classes have been identified, deer are found most often in elite deposits, to the extent that the elites favored deer over most other taxa [56, 91]; middle-tiered social classes had more varied diets by comparison. Several white-tailed deer skeletons were recovered in a large midden in Ceibal’s East Plaza of Group D, an area of the site constructed during the Late Classic period that housed the highest-ranking elite citizens of the community (Fig 10D). Several white-tailed deer were found in the midden behind the Terminal Classic palace in Group A’s East Court as well. These concentrations of deer suggest the animals had been targeted specifically for consumption by the royal elites.

Isotopic data on a subset of these bones, including those found in these elite middens, indicate that none of these deer were consuming high or even moderate levels of maize, ruling out previous theories that deer were raised in captivity in order to support the high demand for their meat during the Classic period [117]. Other isotope studies have found that most deer in archaeological assemblages have C3 diets, meaning they ate little to no maize [124, 125, 133, 134]. While it is still possible some deer were captively raised (see Lagartero [125] and Mayapan [4]), it does not seem to have been a common practice.

#### The diversity of other mammals

There is a large variety of forest-dwelling mammals in the Ceibal assemblages, particularly smaller-bodied taxa, including rabbits (*Sylvilagus* sp.), various opossums (Didelphidae), pacas (*Cuniculus paca*), agoutis (*Dasyprocta punctata*), coatimundis (*Nasua narica*), raccoons (*Procyon lotor*), weasels (*Mustela frenata* and *Galictis vittata*), armadillos (*Dasypus novemcinctus*), and even anteaters (*Tamandua mexicana*). Unfortunately, the majority of these bones did not survive as well as those of larger-bodied mammals, and they occur so infrequently at the site that there is no clear evidence of a pattern over time or space among them. Many of these bones were recovered from primary middens, such as the one located behind the Group A East Court palace, the middens in the Karinel Group, a large animal and ceramic midden on Structure A-2, or the large Group D East Plaza midden (S1 Table). Since very few were found in construction fill from repurposed/secondary refuse, it is likely that the process of using and moving secondary midden material for fill may have destroyed many of these fragile bones.

A few animals appear to have been deposited in special contexts on purpose. One of these was the partial skeleton of a rabbit found near the hand of the aforementioned Middle Preclassic adult male found in Burial 126 from the Jul Group, who also had several other ceramic and shell offerings placed about his body [82]. Fragments of deer antlers were found placed near the skull of human Burial 143, in the Terminal Classic phase of the Amoch Group, which may have been from a headdress [82]. A partial skeleton of a coatimundi was found underneath a Late Classic floor in the Karinel Group. Other animals may have had an important function during life based on their unusual place of deposition. The midden on the slope immediately behind the Terminal Classic palace contained an odd arrangement of species; in addition to several sea urchin spines, other specimens included the mandibles of a margay (*Leopardus wiedii*), the mandibles of a kinkajou (*Potos flavus*), and the arm of an anteater. It is possible these were the remains of costume paraphernalia. Considering there are a number of animal components in the costumes depicted on the Ceibal stelae from the Group A plaza, this would not be surprising.

Larger-bodied mammals are found at the site as well, although unlike the deer, they are fairly uncommon. Peccaries (Tayassuidae) are rarely recovered at Ceibal. In general, peccaries are ubiquitous across Maya sites but are rarely found in high numbers (e.g., [105, 108, 122, 128, 129, 135, 136]). Tapirs (*Tapirella bairdii*) were also rarely found, and the isolated specimens that were occasionally recovered (teeth and phalanges) indicate that they were likely not carried whole to the site, but rather only certain elements. This has been noted at other sites where tapir remains, usually cranial or foot elements, have been found [56, 137, 138]. The Karinel Group had the majority of tapir remains, appearing in both Preclassic and Classic deposits.

Large felines were similarly uncommon and never found as partial skeletons, as was more often the case for the smaller animals. Like the tapirs, their bones were usually found singly, often making it difficult to identify the species if key distinguishing characteristics were missing [139]. Large cat paws and skins are identifiable on many of the Ceibal Late-Terminal Classic stelae, suggesting that at least some of these bones may have been parts of costumes or decorations, which may also explain their isolated recovery at the site. Feline bones are generally uncommon at Maya sites, although they are most frequently found in special and ceremonial deposits, such as human burials and caches [69, 77], perhaps having been deposited as skins or, in rare cases when the entire skeleton is available, as a sacrificial offering [5].

## Conclusions

The Ceibal faunal assemblage is a unique opportunity to examine 2200 years of Maya history through the perspective of faunal trends over time. A uniform excavation strategy covering an area of several kilometers and incorporating both fine-screen and flotation methods produced a large faunal assemblage that offered the chance to address several important questions regarding continuities and discontinuities in the collection, trade, and use of animal resources in the area.

Perhaps the most significant trend at Ceibal is the decline in freshwater mollusks between the Preclassic-Classic transition about 2000 years ago. This same pattern has been noted by other Maya archaeologists, especially in Belize and eastern Guatemala. The fact that the pattern occurs along the Pasión as well indicates that it was a widespread occurrence, involving species from different watersheds. Furthermore, excavations across Ceibal’s ceremonial core, outlying house groups, and the nearby minor center of Caobal reveal that the pattern can be found virtually everywhere humans had lived during the Preclassic. The decline, but never full disappearance, of freshwater mollusks during the Classic period suggests that the invertebrates might not have been entirely depleted, but rather a sociocultural change following the Preclassic was the cause. As settlements continued to grow and become incorporated into full-fledged state systems, perhaps the lack of *Pomacea* and river mussels in middens around the centers of large communities was due to an elite preference for vertebrates. As Platform 97 at Ceibal shows, those living immediately beside the river may have continued to consume shellfish. Invertebrates may have been a minor dietary supplement for the rest of the community, but for the most part, they were replaced by a wider variety of vertebrate taxa as more domestic and husbanded species were introduced on the landscape. While this Preclassic-to-Classic invertebrate trend has been noted elsewhere in the Maya lowlands, the hypothesis that it is linked to the increasingly centralized political organization of the Classic period needs to be examined more thoroughly. Closer examination of the faunal remains from lower-class and rural Classic period households may determine whether it was a pattern unique to the monumental site cores.

The Ceibal assemblage supports two previously hypothesized species introductions from Central Mexico into the Maya lowlands. One of these is the domestic turkey, which appears to have come to Ceibal sometime during the Classic period. The other species is the river turtle, *Dermatemys mawii*, which already existed in the Pasión but appears to have become much more common in the Classic period. The idea that some *Dermatemys* were moved from the Isthmus of Tehuantepec across Guatemala requires strong evidence to substantiate, since natural populations of *Dermatemys* already lived in the region. Further genetic analysis on the archaeological specimens and comparison with the modern populations may solve this mystery.

The dense animal bone middens found near Ceibal’s elite structures suggest there may be a difference in terms of how animals were obtained, used, and discarded among the site core and outlying residential groups. Only the Karinel Group had dense Late and Terminal Classic faunal deposits, but since that group is the closest to Group A’s Central Plaza, it may have housed individuals who had a direct role to play in Ceibal’s ceremonial core. The lack of similar dense vertebrate assemblages in the Preclassic may be due to preservation bias, in that over many centuries, the old middens were recycled and incorporated into structures as fill material. This is likely the explanation for the lack of many thin-boned animals during the Preclassic, particularly birds and amphibians. However, the distribution of mammals, fish, and freshwater mollusks does not seem to vary across Ceibal during the Preclassic period, and many of the same species are found in both the core and the outlying groups. This suggests that faunal resource use was more uniform across Ceibal and its periphery during the Preclassic phases, but during the Late and Terminal Classic there were more distinct differences in how animals were acquired and used.

Tracking the animal resources over time at Ceibal has shown that Maya society’s relationship with the landscape was ever-changing. As political and economic developments altered the southern lowlands, they affected which animal resources people had access to, or which they chose to hunt and fish over others. Vertebrate diversity was always high among the Ceibal faunal assemblage, but for reasons that are still unclear, the Maya shifted their focus from an invertebrate-dominant subsistence base to that focused on fish, turtles, deer, and increasingly, domestic fauna like turkeys. These changes had a lasting impact on the landscape, and perhaps even the ranges of modern fauna today. Future studies at Ceibal and other sites will no doubt provide answers to these questions, which are important for understanding what happened in the past as well as the landscape as we see it today.

## Supporting information

S1 Table List of fauna specimens from Ceibal and Caobal. Specimens are listed according to the excavation operation and bag from which they were found. Relevant notes pertaining to each column, including acronyms, are described in comment boxes in the heading (first row).
(XLSX)

S2 Table Location descriptions at Ceibal and Caobal. This list contains information about the locations for each of the excavation codes in the S1 Table.
(XLSX)

S3 Table Site divisions for minimum number of individuals (MNI). Groups distinguish excavation operations that are found in close enough association that they may contain the same animal remains. Group numbers are arbitrary and only meant to distinguish similar operations.
(XLSX)
