# Correction: The Maya Preclassic to Classic transition observed through faunal trends from Ceibal, Guatemala

**DOI:** 10.1371/journal.pone.0233193

**Published:** 2020-05-08

**Authors:** The PLOS ONE Staff

## Notice of Republication

This article was republished on April 17, 2020, to remove Supporting Information files that were incorrectly included in the originally published article. The publisher apologizes for the errors. Please download this article again to view the correct version.
